# Associative and categorical priming in a word-picture paradigm: a diffusion model analysis

**DOI:** 10.1007/s00426-025-02234-w

**Published:** 2026-02-07

**Authors:** Shanqing Gao, Ines Marberg, Alexander Berger, Andreas Voss

**Affiliations:** 1 Department of Psychology, Heidelberg University, Heidelberg, Germany; 2 Department of Psychiatry, Ulm University, Ulm, Germany

**Keywords:** Word-picture priming, Associative priming, Category congruence priming, Picture processing, Diffusion model

## Abstract

When we perceive language cues, they are processed with a high degree of automaticity and can thus guide the processing of subsequent perceptions. We investigated here how associated and categorically congruent prime words influence responses in a semantic picture categorization task. A hierarchical diffusion model is applied to disentangle the underlying cognitive processes. In the experiment, participants were asked to categorize target pictures as living or non-living. These target pictures were preceded by prime words, for which associations and semantic category match with targets were manipulated. Results indicate robust priming effects of category congruency for both response times (RT) and error rates (ER), whereas associations only show an effect on response times (RT). Diffusion model analysis revealed different cognitive processes for both types of prime-target relations: Specifically, associative priming maps to non-decision times, suggesting a head start in visuo-semantic picture processing, whereas categorical priming was found to affect drift rate, suggesting facilitation of the decision-making process. These results suggest that priming effects in picture classification differ from cognitive processes involved in word-word priming. The implications for theoretical models of priming are discussed.

**Supplementary Information:**

The online version contains supplementary material available at 10.1007/s00426-025-02234-w.

## Associative and categorical priming in a word-picture paradigm a diffusion model analysis

Our perception, interpretation and reaction to events in the world always occur in a context. What we encounter before an event, or the ideas we have just formed, can influence how we process what we subsequently perceive. How the processing of a preceding stimulus (i.e., prime) affects the processing of a subsequent stimulus (i.e., target) is usually investigated in so-called sequential priming studies (e.g. McNamara, 2005; Neely, 1991). Typically, if the prime is related to the target, it facilitates target processing, speeding up responses and reducing errors. There are different mechanisms thought to contribute to such priming effects.

One of the possible contributing mechanisms is spreading of activation from a perceived prime stimulus to associated target concepts. These associations of concepts form in our memory, when we often meet exemplars of these concepts in close temporal relation. For example, we can anticipate rain when we see a dark cloud, because we learned that the two often co-occur. At the same time, sequential priming is not always based on association of concepts in memory. Other explanations of priming effects are rather based on category membership. In this case, we abstract from the individual exemplar and understand it as a member of a certain category. For instance, a fish and a lion are both animals. Identifying a category means highlighting certain features (e.g., animacy) while ignoring others (e.g., the size of the fish or lion).

Priming studies try to tap into the cognitive mechanisms underlying the processing of different types of prime-target relationships. The strength of an association between two concepts can be measured by how often one concept spontaneously brings the other to mind. In practice, this is typically assessed by asking people to report the first word that comes to mind when they hear or read a cue word (Moss et al., 1995). Thus, associative priming is assumed to rely on the facilitation of the retrieving concepts that are strongly tied to a preactivated concept in memory. In contrast, categorical priming is supposedly based on feature overlap between two concepts (for a more detailed distinction between semantic and associative relations, see Moss et al., 1995), such as the shared features of being animals in the case of a fish and a lion.

In categorical priming, where targets in a categorization task are preceded by primes matching or not in the relevant category, two aspects of the relationship between the prime and the target must be taken into account: a semantic similarity relation consisting of feature overlap and a congruency in the dimension to be evaluated in the response. In other words, this paradigm confounds semantic priming with response priming in a categorization task. On the other hand, associative pairs can either share a category (i.e., animacy), such as king–queen, or be linked based solely on co-occurrence, such as king–crown. Therefore, it is possible to combine association and category/response congruence in a 2 × 2 factorial design, as we did in the present study, with a semantic categorization task (living/non-living) in a word-picture priming paradigm.

Surprisingly few studies so far have applied semantic categorization tasks in a word-picture priming paradigm to investigate the contribution of the cross-modal^1^ effect of word primes to picture processing. However, such priming experiments could provide valuable insights into semantic processing of pictures in general and its susceptibility to semantic relatedness with the prime exceeding shared modality. Linguistic priors appear to facilitate perceptual processes, for example, word cues were found to promote perceptual sensitivity of detection in a motion direction task (Meteyard et al., 2007), to speed up image recognition (Boutonnet & Lupyan, 2015), and to facilitate recognition of ambiguous images (Samaha et al., 2018). However, the exact processes by which language primes influence picture processing are still unclear: There are studies suggesting that this influence occurs in early visual processing (Boutonnet & Lupyan, 2015; Samaha et al., 2018), whereas other results indicate an impact on later semantic processing (Francken et al., 2015; Tan et al., 2008).

Diffusion models can further improve the understanding of ongoing processes, as they make it possible to pinpoint potential priming effects on specific cognitive processes (Ratcliff et al., 2016; Voss et al., 2013a). While the effects on reaction time and error rate may appear similar across different studies, these effects are often the result of different processes. Diffusion model analysis is a mathematical tool that leverages distribution patterns in the combination of both reaction time and response data to identify the cognitive processes underlying the observed behavior. It has proven to be a useful tool in the analysis of responses to word stimuli and in tasks involving visual stimuli that do not evoke semantic processing (e.g., Gabor gratings as in Nunez et al., 2019). However, so far, the model has hardly been exploited in paradigms with semantically meaningful pictures as targets. To our knowledge, only one study has investigated word-picture priming in a semantic task with a diffusion model, using a gender categorization task on face stimuli (Todorova & Neville, 2020). In contrast to this study, we independently manipulated two different aspects of the relationship between prime-target pairs, i.e., associations and category congruence, in a semantic categorization task.

In the following, we will review theoretical accounts of the mechanisms involved in associative and categorical priming based on the word-word priming paradigm. We will then highlight potential differences between theories of word priming and word-picture priming. After that, we will discuss how diffusion modelling can be used to elucidate the processes involved in priming.

### Theoretical accounts of associative and categorical priming

The term “semantic priming” is used more often in studies than the terms “associative priming” and “categorical priming”. Semantic priming refers to the priming effect that comes from semantic relations of prime and target stimuli, which might comprise semantic similarity, associative relationships, or both (McNamara, 2005). Semantic similarity can be defined as the degree of feature overlap between two concepts. For example, the concepts ‘couch’ and ‘armchair’ share many features, as they are both pieces of furniture, are designed for sitting, have soft coverings, and so on. As previously mentioned, semantic association refers to the likelihood that one concept (or word) will bring the other concept (or word) to mind. For example, ‘dog’ might be the first thing that comes to mind when someone reads the word ‘cat’. This example shows, that associated concepts have often – but not necessarily – some feature overlap. Although semantic similarity and semantic association do not always occur together, highly associated concepts tend to share more semantic features than less associated concepts (Hutchison, 2003; McNamara, 2005). This may explain why word pairs that are highly associated and belong to the same semantic category produce strong and reliable priming effects. However, it has been demonstrated in a meta-analysis (Lucas, 2000) that pure semantic similarity (i.e. based solely on category membership, in the absence of associative links) also produces reliable priming effects, although this effect was smaller compared to the mixture of semantic similarity and association.

A large body of research has analyzed semantic priming effects with words as prime and target stimuli using different experimental tasks like lexical decision, naming, or semantic categorization (Wentura & Degner, 2010). Although there are some important differences between the tasks, the basic underlying mechanisms are often assumed to be similar. Spreading activation theory (Collins & Loftus, 1975) is arguably one of the most widely used accounts to explain the effects of semantic priming, which is seen as an automatic process that can occur rapidly without intention or awareness. Collins and Loftus (1975) proposed that semantic concepts are interconnected in semantic memory. Thus, when one concept node is activated, this activation spreads to other related (i.e., associated) concept nodes. The closer and stronger the associated nodes are connected to the activated concept, the more activation they will receive. For associative priming, the spreading activation model is the dominant account: It is assumed that target identification is facilitated, if the target concept nodes are pre-activated by the prime, the retrieving of target information from memory is facilitated, resulting in faster and more accurate responses to associated than to unrelated targets.​​.

In unitary concept node models, such as the spreading activation account, associations between nodes (concepts) must be learned individually through experience. In contrast, the distributed memory model (Masson, 1995) proposes that concepts are represented as activation patterns over multiple semantic features. Semantic relationships emerge from feature overlap, though the model also allows for learned associations that do not rely on such overlap. In this view, priming occurs as a natural consequence of overlapping activation patterns between related primes and targets, supporting a natural explanation for categorical priming.

Although these two memory models differ in how they represent semantic information, both assume that prime presentation facilitates target identification via semantic pre-activation^2^. Our goal in this study is not to adjudicate between them, but to present them as complementary explanations for the priming mechanisms. Other strategic models to explain semantic priming effects, such as expectation generation (McNamara, 2005; Neely, 1989) or retrospective semantic matching (Neely et al., 1989), are less relevant to the picture targets used in this study and will therefore not be discussed here.

While both associative and categorical priming can involve (automatic) semantic pre-activation mechanisms, categorical priming in semantic categorization tasks often involves additional *response congruence*, where the locus of priming occurs at the stage of response selection or execution (De Houwer et al., 2002; Klinger et al., 2000; Van den Bussche et al., 2009a, b; Voss et al., 2013b).^3^ In these tasks, participants must make binary decisions about target category membership (e.g., *living* vs. *non-living in the present study*), and the prime either matches or mismatches the target’s category. Category priming has been demonstrated in tasks with a wide variety of categories, such as numerical judgements (Dehaene et al., 1998), gender classification (Todorova & Neville, 2020), valence assessment (i.e., affective priming; Klauer & Musch, 2003), object size judgements (Kiesel et al., 2006), and semantic category classification (Ortells et al., 2016).

Response priming can be explained by the implicit application of a given task set — structured links between specific instructions and their associated actions that are formed and maintained in procedural working memory (Oberauer et al., 2013). It is assumed that these task sets, which are relevant for target stimuli, are also (consciously or unconsciously) activated by the perception of prime stimuli (Ansorge et al., 2014; Kiefer & Martens, 2010). In a categorization task, for example, a task set could comprise the rule to press the left key for animals and the right key for non-living objects. When this task set is applied to a prime stimulus, it is implicitly categorized as living or non-living, and the corresponding response code is activated (although overt responses are typically not executed). This pre-activation of response codes may subsequently facilitate or interfere with responding to the subsequent target. This view is consistent with theoretical accounts that conceptualize task sets as operating at the categorical or decision level of response selection (Logan & Gordon, 2001; Vandierendonck et al., 2010; Klauer et al., 2007), rather than at the level of concrete motor preparation and execution.

This interpretation aligns well with the response facilitation/competition account mentioned in earlier literature (also referred to as the Stroop-like mechanism in Klinger et al., 2000; De Houwer et al., 2002; Klauer & Musch, 2002). In particular, this account argues that, in congruent trials, primes pre-activate responses that align with the target response, leading to faster response. In contrast, in incongruent trials, primes pre-activate responses that conflict with the target response, causing interference and thus delaying the response. Such response facilitation/competition can occur at different representational levels, including abstract response codes at the decisional stage and response preparation and execution at the motor stage, depending on experimental design and task demands. Whether prime-induced pre-activation at a semantic level can additionally lead to pre-activation of a motor response, and under which conditions, is still the subject of ongoing research. Voss et al. (2013a, b) proposed that this is what occurs in categorical priming in word categorization. Moreover, recent research posits that activity in motor areas of the brain may already be building up during decision-making processes (e.g., Servant et al., 2021; Dendauw et al., 2024), but these observations, to the best of our knowledge, have not been linked to priming so far.

Direct motor activation (i.e., bypassing semantic processing) may also occur, but it seems to be limited to specific cases. One such mechanism involves stimulus-response (S-R) associations that are learned during an experiment in which primes previously served as targets (Abrams et al., 2002; Dehaene et al., 1998; Damian, 2001). Secondly, the action-trigger theory (Kunde et al., 2003) suggests that participants can anticipate complete sets of possible stimuli and prepare corresponding motor responses, so that matching primes automatically trigger action preparation, even for novel primes. However, this is only feasible when the task demands involve a small target set size or a narrow category (e.g., four possible items or single-digit numbers), and is unlikely to operate when tasks involve a large set of possible targets or broad semantic categories (e.g., “living” vs. “non-living”; Micher & Lamy, 2023).

To sum up, both purely semantic and response-related mechanisms are able to explain categorical priming. Purely semantic mechanisms are based on spreading activation or automatic activation by feature overlap. They are thought to be observable independently of the task. For example, the shared feature of animacy would also affect processing speed in a task that is orthogonal to the animacy distinction (e.g., affective categorization), if the shared feature induced automatic activation of possible other referents with this feature. However, in semantic categorization tasks, evidence for purely semantic processing has rarely been found, possibly because stimuli were selected from broad categories and the semantic distance was still large (e.g., trout–elephant; de Wit & Kinoshita, 2015; Quinn & Kinoshita, 2008; Van den Bussche et al., 2012). Ortells et al. (2016) controlled for semantic distance by comparing the influence of strongly and weakly semantically related novel primes on subliminal categorical priming using broad target sets (e.g., animals vs. body parts). They found that the priming effect modulated the N400 ERP component – an index of semantic activation (Kutas & Federmeier, 2011) – only when the primes and targets were strongly semantically related. On the other hand, the response-related mechanisms would depend on the task and produce an effect via the mechanism of response facilitation/competition, which induced by a task set or by purely response-based mechanisms like action triggers or S-R bindings. When task set is applied implicitly, features that are relevant to the task are highlighted, thus semantic processes should be involved if a priming effect is observed.

### Word-word vs. word-picture priming

The literature reviewed in the previous paragraphs is mainly based on data for paradigms using words for primes and targets, whereas the present study employs picture targets. Priming and related decision-making processes have been extensively studied for words, and several computational models have been developed to simulate the access to semantic memory through language (Kumar, 2021). However, less is known about how picture priming works and how visual input informs the decision-making process. Concept nodes of the spreading activation account could arguably be identical for words and pictures with the same referent, differing only in their modality of activation. Several early studies have suggested that a common semantic representation could be accessed alike by either a word or a picture (e.g., Kroll & Potter, 1984; Sperber et al., 1979), and cross-modal priming studies have corroborated these findings by demonstrating that using stimuli of the same modality is not necessary for priming (Carr et al., 1982; Irwin & Lupker, 1983; Spruyt et al., 2002). Nevertheless, even if we assume that there are shared concepts between words and pictures (either as unitary nodes or as a distributed activation pattern), there are differences in how concepts are accessed via words and pictures. When decisions are based on words, it is not possible to select the response directly from the visual properties of the stimulus. As a symbolic representation, a word must go through the orthographic and/or phonological levels and the lexical entry must be identified before accessing the semantic level. Note that lexical access (i.e., confirming that a word is part of the lexicon) and accessing its phonological representation is in principle sufficient for tasks like lexical decision or naming, which are commonly used in priming. Thus, semantic processing in these tasks may remain shallow, and observed priming patterns and processes might therefore differ in word-picture paradigms.

Pictures, on the contrary, are non-symbolic representations. Compared to words, pictures have been shown to produce faster responses in semantic categorization tasks (termed the picture superiority effect, see, e.g., Irwin & Lupker, 1983; Taikh et al., 2015; Potter & Faulconer, 1975), and therefore are often assumed to access the concept nodes of the semantic system more directly. However, picture processing still requires the construction of a meaningful representation of the raw input via analysis and binding of visual features like shape and color, and the matching to visual schemata (e.g., knowledge what a dog looks like). Our perceptual system is very efficient in this process and does not necessarily require much visual input to produce valid results. Initial gist processing may already be sufficient for superordinate and basic level categorization, especially if speed is favored over accuracy or no further visual uptake is possible (Clarke et al., 2011; Fabre-Thorpe, 2011). For example, you do not need to know that a picture shows specifically a dog in order to recognize that it shows an animal. Shape information or the presence of eyes can already be diagnostic and support successful ultra-rapid categorization. However, when there is no pressure to classify rapidly, semantic or cross-modal integration processes might gain more weight. Thus, pictures provide multiple access routes to associated knowledge, not only through identification of the depicted object’s name and associated semantic knowledge, but also through its shape and other visual features.

Priming the perception of pictures with word primes could have an influence, as more and more studies conclude that verbal cues can modulate this process from early stages on (see, e.g., Gilbert & Li, 2013; Wolfe, 2021; Simanova et al., 2016; Lupyan et al., 2020). In general, conceptual and perceptual processing seem to be highly intertwined in visual processing, but many questions are still open in this regard.

Moreover, changing the modality of the target stimulus presentation (text words vs. pictures) could also affect the association relationships. Associations are often based on co-occurrence of concepts in experience (Stacy et al., 2006). Importantly, memory concepts can be activated by perceptions in different modalities. For example, the activation of a concept like “apple”, can result from a visual image of an apple, its smell, its touch, or reading or hearing the word itself. However, which features of a concept are activated depends on the modality as well as the context (Truman & Kutas, 2024; Saffran et al., 2003), and thus modality might affect the triggered associations. As association norms are usually collected based on words, they might partly reflect lexical associations due to frequent co-occurrence in texts. It is difficult to determine what associations a word elicits in other modalities such as the visual domain.

Regarding the mechanisms of categorical priming, animacy is a central feature of objects and is likely to be consistently activated across modalities. However, as explained earlier, it may be accessed differently because flexible access to pictures may be susceptible to preceding linguistic cues in a different way compared to words. In terms of response-related mechanisms, a differentiated pattern emerges in cross-modal settings. Specifically, action triggers are unlikely to be established when the expected target modality does not match the prime modality (Van den Bussche et al., 2009a; Van den Bussche et al., 2012). However, task-set execution may still play a role if participants maintain a modality-independent mapping between categories and responses (e.g. “living” -– left, “non-living”– right). Consequently, a priming effect in cross-modal setting is expected to be rooted in amodal or multimodal semantic processing, while purely response-based mechanisms are unlikely.

### Assessing priming mechanisms by diffusion modeling

The present study aims to clarify the cognitive processes underlying priming by applying diffusion model (DM) analysis (Ratcliff & McKoon, 2008; Voss et al., 2013a). Beyond the theoretical accounts discussed earlier, DM analysis provides a perspective at the level of algorithms (Marr, 1982) that can be used to better understand the mechanisms driving priming effects. By modelling response times and binary decisions, the DM enables us to draw inferences about the specific cognitive processes involved in tasks such as the living/non-living classification used here.

#### The diffusion model rationale and its parameters

**Fig. 1:**
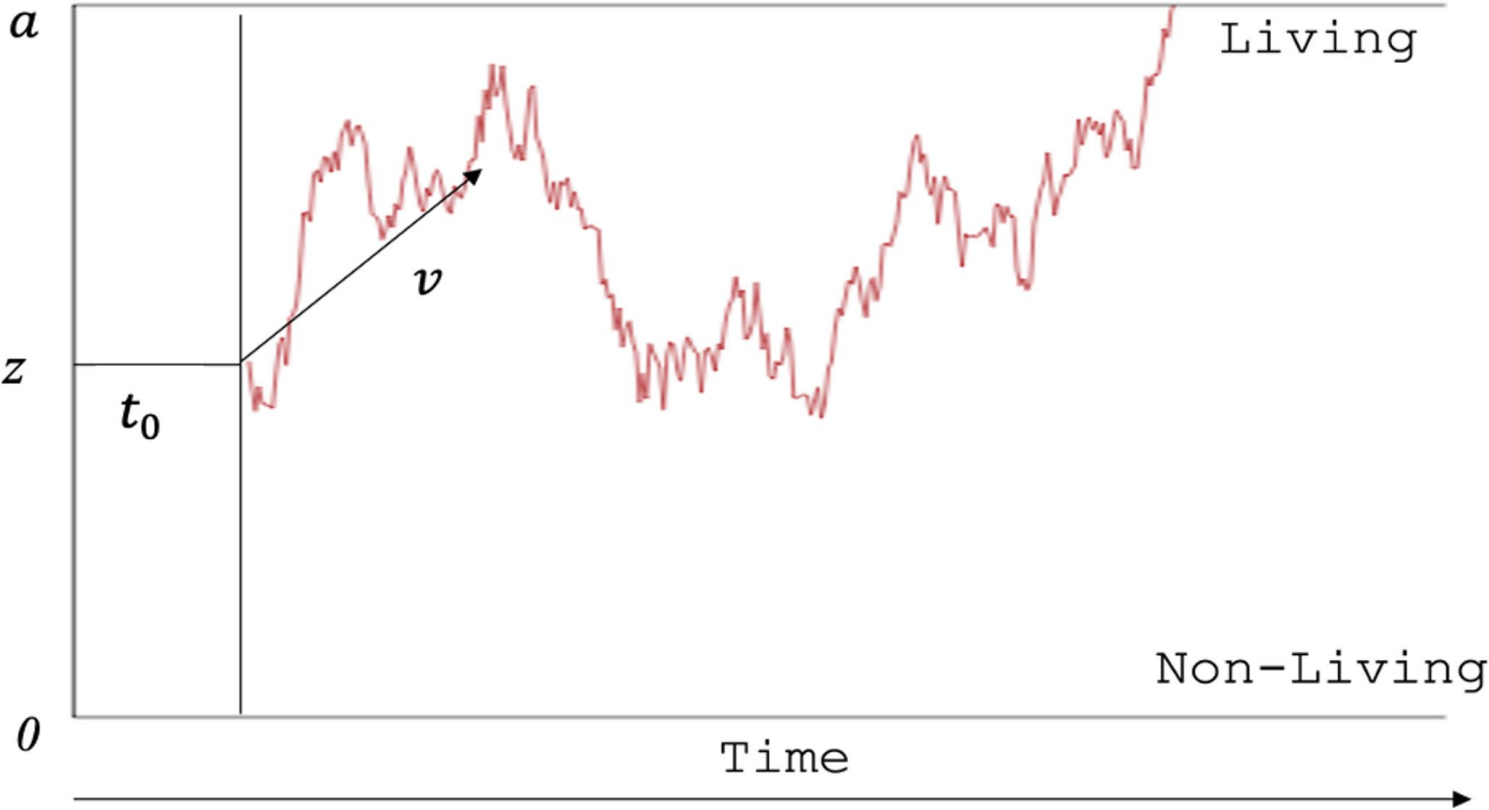
A graphical illustration of the diffusion model. *Notes.* The decision process starts from the starting point (*z*) with a constant drift (*v*) and runs over time until it hits the upper bound (*a*) or the lower bound (0). The jagged line is an example of the latent path of evidence accumulation in one trial of the experiment. Once a threshold is hit, the corresponding motor response is triggered

The basic DM assumes that decisions are based on a continuous stochastic process of evidence accumulation, which starts from an initial point (the starting point, *z*) and continues until it reaches one of the two decision boundaries. These boundaries represent the two possible responses in a binary decision task. When the evidence reaches one of the thresholds, a decision has been formed and the corresponding motor response is initiated. Figure 1 illustrates this decision process, which is preceded by a stimulus encoding phase. Note that also post-decisional motor processes contribute to the non-decision time ($\:{t}_{0}$), which are, however, not shown in the figure. Although it shows a single example of an evidence accumulation trajectory, it is important to note that this is just one of many possible paths. The accumulation process is inherently noisy^4^, indicating that even when the same stimulus is presented on different trials, the trajectory of evidence accumulation will not be identical. This leads to different decision times and even different responses for every single trial.

The drift rate (*v*) reflects the average speed and direction of evidence accumulation. An average slope of information uptake towards the upper (lower) threshold is indicated by positive (negative) drift rates. Higher absolute values of the drift rate indicate higher speed of evidence accumulation resulting in faster RTs and increased accuracy. Drift rate tends to be higher for easy tasks than for difficult ones (Voss et al., 2004) and is associated with higher intelligence and greater working memory capacity (Lerche et al., 2020; Ratcliff et al., 2010; Schmiedek et al., 2007).

Boundary separation (*a*) maps the amount of information required to make a decision and is therefore a measure of the participant’s decision-making strategy. Large (small) threshold separations indicate conservative (liberal) decision settings, and lead to slow (fast) responses with high (low) accuracy. Numerous studies have demonstrated the sensitivity of threshold separation to speed versus accuracy instructions (e.g., Voss et al., 2004). The starting point (parameter *z*) represents a potential decision bias. If *z* is biased towards one of the thresholds, the corresponding responses will be chosen more often and will be faster on average. The relative starting point (*w* = *z/a*) reflects whether the decision is unbiased (*w* = 0.5) or biased towards one of the two response alternatives. Besides the decision process, the duration of stimulus encoding (prior to evidence accumulation) and motor planning and execution (after a decision has been made) are captured by the non-decision time parameter ($\:{t}_{0}$). The DM assumes that these processes occur in sequence, rather than overlapping in time.

#### Associative priming in diffusion model analysis

Several studies have investigated the cognitive underpinnings of priming effects in word paradigms using DM, but the results remain inconsistent. Voss et al. (2013b) compared processes of associative priming and categorical priming. For associated prime-target pairs, an increase in drift rates during the decision process was observed, suggesting a facilitation in target accessibility. This finding aligns well with the spreading activation account, whereby pre-activation of the target supports its retrieval from lexical memory leading to faster and easier identification of the target. The effect of prime-target associations on drift rates was observed both in the lexical decision task and the semantic categorization task (Voss et al., 2013b; Experiments. 1a, 3a and 3b), which suggests that the spreading activation mechanism in associative priming is independent of task type.

Less clear are effects of associative priming on non-decision time. The authors report that associated primes —in addition to the effect on drift rate—also reduced non-decision time in the lexical decision task (Exp. 3a). However, this effect was not observed in the semantic categorization task– a task, which effectively rules out potential influences of post-lexical processing. The authors argue that in a lexical decision task, post-lexical backward matching could contribute to the observed effect on non-decision time (also see Kiefer et al., 2023). After subjects have recognized the target word, it is evaluated retrospectively whether the prime is associated with the target. This is because only the word targets are preceded by associated primes, whereas the non-word targets can never be preceded by associated primes. Similarly, Gomez et al. (2013) reported associative priming effects on both drift rate and non-decision time in a lexical decision task with clearly visible primes, whereas only the effect on non-decision time remained with masked primes. However, they proposed an alternative interpretation of the possible process represented by the effect on non-decision time especially in the context of masked priming – as a pre-decision perceptual processing of the target. During the initial stage of perceptual information processing, incoming information must first be encoded, and only after this encoding is complete can it inform evidence accumulation. This effect at the early stage is referred to as a head start in target processing. We think this mainly refers to low-level visual/lexical processes (i.e., recognizing the orthographic features). A similar effect has also been found in other studies using the lexical decision task: a robust associative priming effect on drift and a weaker, sometimes non-significant, effect on non-decision time (Berger et al., 2021; Lerche & Voss, 2017).

#### Categorical priming in diffusion model analysis

As discussed in the theoretical section, category priming effects can arise from either purely semantic or response-related mechanisms (De Houwer et al., 2002; Klinger et al., 2000; Klauer & Musch, 2002). These two mechanisms are not mutually exclusive and may jointly contribute to category priming effects (Kiefer et al., 2015). Within the diffusion model framework, response-related category priming effects can be theoretically mapped on decisional process and post-decisional process, depending on experimental design or task demands.

Voss et al. (2013b; Exp. 1b, 2a and 2b) observed categorical priming effects at the non-decision time ($\:{t}_{0}$) parameter. Specifically, on-decision times were shorter for category-congruent prime–target pairs than for incongruent pairs, suggesting that response execution was faster in the congruent condition. Beyond the $\:{t}_{0}$ parameter, Voss et al. (2010) introduced another parameter, *d* (*d* = $\:{t}_{0,lower}\:$-$\:{t}_{0,\:\:upper}\:$) to capture response execution biases in categorical priming more directly. Importantly, when this parameter was included in the model, the starting point (*z*) was fixed across conditions. Thus, the model included only one type of bias, namely response execution bias, rather than both response and decision biases. In their model, the upper threshold denoted the correct response, and the lower threshold represented the incorrect one. In the congruent condition, faster execution of correct responses was indicated by a positive *d*, while in the incongruent condition, incorrect responses were executed more quickly than correct ones, as suggested by a negative *d*. This can be interpreted as indicating response facilitation/competition at a post-decisional stage.

Notably, Experiment 2b of Voss et al. (2013b) also employed a semantic categorization task similar to our study. Here, category priming effects were found both for response times and error rates. However, no effect of semantic congruence on the drift rate was observed. Since changes in non-decision time influence only response latencies but not accuracy, they cannot explain the observed impact of primes on error rates. Therefore, additional effects of categorical priming on decision-related parameters (i.e. drift rate and/or starting point) have to be assumed. Possibly, an effect on drift rate was not observed because it was too small to reach statistical significance. Also, as mentioned before, the starting point (*z*) was fixed when the *d* parameter was included in the model, so potential decision biases were not modeled. Supporting this view, a more recent study by Todorova and Neville (2020) using a gender classification task found that the congruence between stereotype-based word primes (e.g., tie, mascara) and face targets influenced both the drift rate and non-decision time, suggesting that both early semantic processing and response-related mechanisms contributed to the observed priming effects. An additional potential parameter involved in categorical priming is the starting point of the decision process. Congruent primes may shift the starting point closer to the boundary associated with the expected primed category, thereby introducing a decision bias. For example, a mood congruence effect in affective categorization could be explained by a decision bias (White et al., 2018).

In summary, previous literature has consistently found that associative priming maps on drift rates, which supports a facilitation on lexical or semantic accessibility. Findings regarding the impact of associated primes on non-decision time have been mixed and may depend on the task. Those effects are often interpreted as post-decisional matching or check mechanisms, or are attributed to facilitation at the level of perceptual encoding. For categorical priming, however, there is no consensus on which diffusion model parameters are impacted by prime-target congruency. While categorical priming has been associated with faster non-decision time—supporting a motor response-based account— theories suggest that semantic processing may also play a role, potentially manifesting increased drift rates. Furthermore, it remains to be tested whether categorical priming may also be related to the starting point of the decision process (i.e., a decision bias). Notably, with the exception of Todorova and Neville (2020), most prior studies have investigated these effects exclusively in unimodal word-based paradigms, without incorporating cross-modal designs involving pictures. Given the distinct processing characteristics of visual images compared to words, it remains unclear whether the mechanisms underlying associative and categorical priming differ in word–picture paradigms from those observed in purely linguistic priming tasks.

### The present study

We aim to compare the cognitive processes underlying associative and categorical priming effects in a semantic categorization task using word primes and picture targets. To uncover the cognitive processes involved, we employ a diffusion model (DM) analysis. In the current experiment, participants were asked to categorize target pictures as either living beings or non-living objects. Each target was preceded by a prime word, with associative and categorical relationships between primes and targets systematically counterbalanced across trials. Certain assumptions of priming theories, specifically the notion of a shared semantic/conceptual representation across modalities, and early experimental results of picture-word priming studies mentioned in the previous sections, suggest that associative and categorical priming effects are generally unaffected by the modality of the stimuli. We predict that prime-target associations and category matching will both facilitate responses; in other words, we hypothesize that we will observe faster response times and/or lower response error rates in the behavioral results for both associated prime-target pairs and prime-target pairs sharing the same category.

With respect to the parameters of the diffusion model we suppose that associative priming involves semantic pre-activation instead of purely lexical processes and thus expect to replicate previous findings: Associated primes should facilitate target processing, reflected in increased drift rates. However, even if words and pictures share access to common semantic representations, pictures may engage these representations through multiple processing routes. Therefore, it remains an open question whether cross-modal presentation alters the nature of associative priming. In addition, it is still unclear whether primes in word–picture paradigms affect early encoding stages, which would be reflected in changes to the non-decision time parameter. Previous findings are inconsistent as to whether non-decision time is affected by prime-target associations, so we refrain from formulating clear hypotheses for the effect of associations on non-decision time.

If a categorical priming effect is observed, it is typically assumed to stem from either response-related mechanisms or semantic processing, as suggested by previous theoretical accounts and empirical findings. In the current experimental design, the picture targets belong to broad semantic categories and comprise a large target set, with each item never serving as a prime. Consequently, specific stimulus–response mappings or action-trigger mechanisms are unlikely to be involved, and purely response-based priming should be minimal. We therefore expect that category congruence between prime and target will primarily affect drift rate rather than non-decision time, indicating that cross-modal categorical priming is driven by semantic pre-activation. However, empirical results by Voss et al. (2013a, b) did show an effect of category match on non-decision time, therefore we also consider this possible, while the mechanism behind would need to be elucidated further. Furthermore, we will also explore whether category-congruent primes bias the relative starting point of the decision process toward the response associated with the prime’s category, thereby requiring less evidence for the target to trigger a decision. While this parameter is often fixed in prior studies (Voss et al., 2013b; Todorova & Neville, 2020), we allow it to vary to test for potential response bias effects of categorical priming.

## Method

### Participants

Forty-nine participants (age: M = 25, SD = 8; 26 female, 23 male) were recruited via the Prolific platform (https://www.prolific.co/). To detect a medium effect size (Cohen’s f = 0.25) in a repeated measures design with a power of (1-β) = 0.90, 44 participants are required (Faul et al., 2009). We oversampled with 5 participants, as in our experience typically data from around 10% of participants have to be discarded because of lack of compliance in online studies.

All participants were English native speakers. Participants received a payment of 1.50 GBP (approx. 2 US$) for completing the 10-minute study. All participants were required to give informed consent before the online experiment started.

### Design and materials

The present experiment included the within-subject factors of category congruence (congruent vs. incongruent) and prime-target association (associated vs. non-associated). As targets, 44 color pictures (22 objects and 22 animals) were selected from a set of Snodgrass and Vanderwart-like objects (Rossion & Pourtois, 2004). To control for target frequency, we analyzed lexical frequency of the animal or object names^5^. Average logarithmized frequency was 2.96 for animals and 3.08 for objects (data from a text corpus based on American subtitles; cf. also Ratcliff, Thapar et al., 2010).

Each target was paired with an associated prime stimulus from congruent and incongruent categories each. For example, for the picture of a tiger, the words lion and jungle were used as the associated primes from the same and different categories. Associated prime words were selected based on the SNOW-EN R123 dataset by De Deyne et al. (2019). Associated prime words had association strengths between 0.1 and 0.3 with the corresponding targets. Mean association strengths were 0.18 and 0.14, for the living targets and non-living targets, respectively (*t(78) = 1.78*, *p* =.08), and 0.18 and 0.13 for congruent and incongruent category pairs (*t(78) = 2.60*, *p* =.01). The concepts of prime stimuli were never repeated as target stimuli (i.e., we always used novel primes). For the non-associated primes, we re-paired the associated prime-targets within each category to generate primes belonging to the congruence category. Finally, we shuffled the associated primes in the opposite category to create the non-associated, category-incongruent primes.

In total, the experiment consisted of 160 experimental trials and 16 practice trials for each participant (see Supplementary Material A), which were presented in random order. Each target picture was used four times with four different primes, ones for each condition. For example, the target picture of a tiger was paired with the words “lion” (associated, same category), “witch” (non-associated, same category), “jungle” (associated, different category), and “pedal” (non-associated, different category).

### Procedure and apparatus

The online experiment was programmed on lab.js (Henninger et al., 2022). Data was collected online through the participants’ browsers, but the experiment ran in full screen mode. Before the experiment started, we collected their personal data, including age, gender, and occupation. They then read the instructions. Participants had to classify target pictures as living or non-living as quickly and as accurately as possible by pressing the response keys [A] and [L], respectively, on their keyboard. The assignment of keys to response categories (living vs. non-living) was counterbalanced across participants. Participants first completed a short practice block (16 trials). Then, instructions were repeated, before the main experiment (160 trials) started.

Each trial began with the presentation of a fixation cross in the center of the screen for 500ms, which was then replaced by the prime word in lower case. After an SOA of 500ms, the prime stimulus was replaced by the target picture. The target remained on the screen until participants made their response. After an inter-trial interval of 300ms, the next trial started.

### Data pre-treatment

Data from 4 participants had to be excluded, because they did not finish the experiment, and data from an additional participant was excluded due to technical problems in data recording. Dealing with outliers in advance is important because outliers can lead to biased parameter estimation, although the best way to remove outliers is still a matter of debate (Berger & Kiefer, 2021; Miller, 2023). Firstly, we excluded trials with response times below 200ms and above 3 s. This step excluded 5.91% of the trials. We then applied Tukey’s (1977) outlier criteria to logarithmized RT data^6^ for each individual separately, that is, logarithmized response times that are more than 1.5 interquartile-ranges below the first quartile or above the third quartile were discarded (3.98% of data).

### Behavioral analysis

Response times (RTs) from trials with correct responses were analyzed using linear mixed effects models (LMM) and response accuracy (i.e., correct vs. incorrect responses) was analyzed using generalized linear mixed effects models (GLMM). Because the experimental design included more than three within-subject factors as predictors, mixed models take into account the dependencies in the data structure and thus provide more accurate estimates of the effects in repeated-measures than traditional analysis. The LMM/GLMM analyses were conducted with the afex package (Singmann et al., 2023), which includes a function mixed() that is built on top of lmer()/glmer(). The p-values for the LMMs were calculated using the Satterthwaite method. The package automatically did sum contrast coding for independent variables.

In the chosen LMM, with RTs as dependent variable, we included category congruence (incongruent vs. congruent), association (non-associated vs. associated), and target type (living vs. non-living), as well as all interactions between these variables as fixed effects. In this experiment design, both category congruence and association were within-subject and within-item factors, while target type was within-subject but not within-item. We started from the “maximal model” (Barr et al., 2013) with crossed random effects. Then, we stepwise simplified the model by removing correlations between random effects within subject and item groups until no overfitting or other warnings returned. ​​The final model included by-participant random intercepts along with random slopes for the predictors category congruence, association, and target type, as well as for the two interaction terms associated × target type and category congruence× target type. The correlations between these random effects were removed to avoid overfitting. At the item level, we included random intercepts, as well as a random slope for category congruence, again without correlations. A comparison between the final and maximal models showed that the pattern of significant and non-significant fixed effects remained consistent, indicating robustness of the results (Supplementary Material B Table S2).

As each target appeared (but paired with different primes) four times within each subject, we fitted the second LMM including target repetition times as an additional factor to assess its potential influence on priming effects. These statistical results are preliminary tests of dynamic processes of priming effects (see Discussion). As the results are not directly relevant to our primary hypotheses, they are reported in Supplementary Material C.

For the analysis of the accuracy data, we followed the same steps as for the RTs analysis. The binary outcome variable was modeled using a GLMM with a binomial distribution and a logit link function. The fixed effect included the same predictors as in the RT analysis. Similarly, we began with a maximal random-effects structure and gradually simplified it to improve model convergence and avoid overfitting, ultimately selecting a more parsimonious model. This final model included a by-subject random intercept and random slope for category congruence, and a by-item random intercept. The model was estimated via maximum likelihood using the Laplace approximation. To evaluate the statistical significance of fixed effects, we used likelihood ratio tests (LRTs), in which the full model was compared against reduced models omitting each term of interest. These comparisons were carried out using the method = “LRT” option in mixed(). The maximal model and the chosen model yielded highly similar fixed-effects estimates (Supplementary Material B Table S3). Notably, the main effect of category congruence and the two-way interaction category congruence× target type remained significant across models. The three-way interaction category congruence× target type × association showed consistent coefficient estimates across models, although the effect remained marginally significant or non-significant in each model.

### Diffusion model analysis

Many previous studies applying the DM to analyze priming effects used a two-step approach: In the first step, parameters were estimated for each subject. These estimated parameters were entered in statistical tests in the second step. This approach can be problematic. First, it requires a large number of trials per subject in each experimental condition (Wagenmakers, 2009). Second, it ignores random effects between subjects and the uncertainty of individual estimates, as the second step treats point estimates as fixed values (Boehmet al., 2018b). In comparison, the estimation efficiency of the hierarchical model is better because the individual effects shrink towards the group mean. In addition, the hierarchical modelling approach is more suitable for small-scale experimental designs, as it can reliably recover individual differences even with relatively few trials per participant (Ratcliff & Childers, 2015).

For the present study, hierarchical DM analyses were implemented using RStan (Stan Development Team, 2024), which allows for Bayesian inference via Markov Chain Monte Carlo (MCMC) sampling. Response coding (as opposed to accuracy coding) was used, with the upper (lower) threshold indicating living (non-living) responses (i.e., different drift rates were estimated for living vs. non-living targets). RTs were entered in seconds (rather than milliseconds), so that the estimated non-decision times ($\:{t}_{0}$) and other time-based parameters are also on the scale of seconds.

We started with a baseline model, that comprises 5 parameters^7^: Threshold separation (*a*), relative starting point (*w*), two drift rates for the two responses ($\:{v}_{living}$, and $\:{v}_{non\:living}$), and non-decision time ($\:{t}_{0})$. Thus, the baseline model ignores all priming effects. In a series of subsequent models, we allowed for an impact of prime-target association status, prime-target category match, and interactions of both factors on drift, non-decision time and starting point (Table 1). Weakly informative priors were chosen for all parameters (see Supplementary Material D). For each model, we ran four MCMC chains of 5000 samples each, half of which were discarded as burn-in. Thus, posterior estimates were based on 10,000 valid samples.

**Table 1:** Different diffusion model variants and corresponding LOOIC values

Model	Category Congruence	Association	Interaction effects	LOOIC
Model 0				−8600.2
Model 1		$\:{v}_{a}$		−8610.2
Model 2		$\:{{t}_{0}}_{a}$		−8671.1
Model 3	$\:{v}_{c}$			−8911.9
Model 4	$\:{{t}_{0}}_{c}$			−8633.0
Model 5	$\:{w}_{c}$			−8823.9
Model 6	$\:{v}_{c}$	$\:{{t}_{0}}_{a}$		−8978.9
Model 7	$\:{v}_{c},{{t}_{0}}_{c}$	$\:{v}_{a},\:{{t}_{0}}_{a}$		−9016.0
Model 8	$\:{v}_{c},{{t}_{0}}_{c}$	$\:{v}_{a},\:{{t}_{0}}_{a}$	$\:{v}_{i},\:{{t}_{0}}_{i}$	**−9087.9**
Model 9	$\:{v}_{c},{\:w}_{c},\:{{t}_{0}}_{c}$	$\:{v}_{a},\:{{t}_{0}}_{a}$	$\:{v}_{i},\:{{t}_{0}}_{i}$	−8693.1

*Notes. v*, $\:{t}_{0}$, and *w* denote that the model assumes effects of experimental manipulations on drift, non-decision-time, and relative starting point, respectively. Indices $\:a$, $\:c$, and $\:i$ denote that the corresponding parameter may differ depending on prime-target associations, category congruence, or on an interaction of both factors. LOOIC is the information criterion of leave-one-out cross validation, with smaller values indicating better model fit

Model comparison was performed using leave-one-out cross validation (LOO-CV) to select the model that best fitted the data (Vehtari et al., 2016, 2023). Smaller LOO Information Criterion (LOOIC) values indicate better goodness of fit. LOOIC has displayed more robust performance than other information criteria like WAIC when there are outliers in observed data, and it is less influenced by the choice of priors. In addition, LOOIC is suitable for comparing models with hierarchical data structures (Vehtari et al., 2016).

## Results

### Behavioral results

**Fig. 2:**
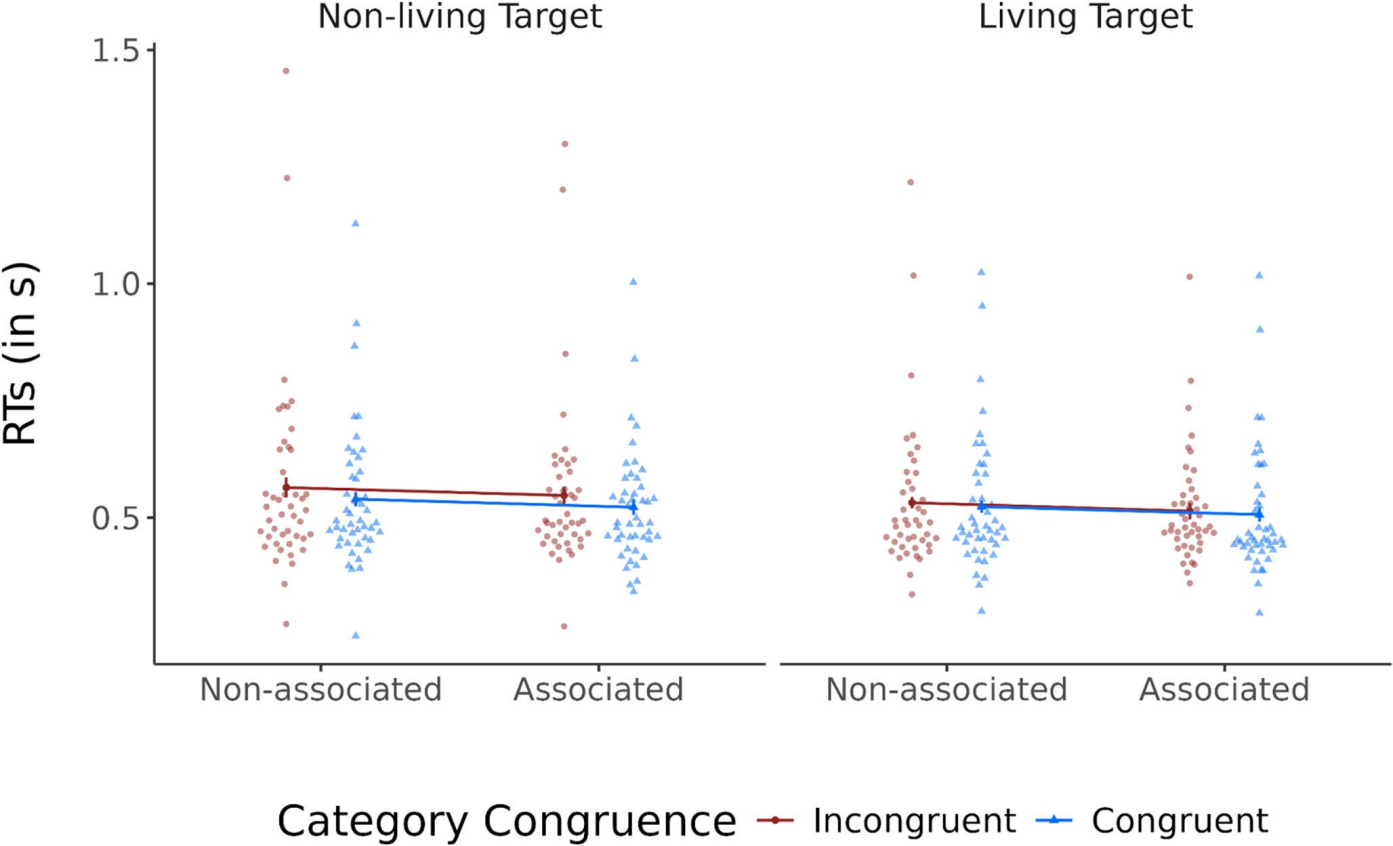
Correct RTs (s) as a function of semantic association, category congruence and target type. *Notes: *Points show individual participant means, lines show values averaged across participants, and error bars denote 95% within-subjects confidence intervals of the means

A linear mixed model analysis on RTs of correct responses (for a visualization, see Fig. 2) showed main effects of semantic association, β = 0.009, SE = 0.003, *t*(24.58) = 3.27, *p* =.003, and of category congruence, β = 0.008, SE = 0.003, *t*(34.93) = 2.67, *p* =.011, indicating faster RTs in trials with associated primes and with category congruent primes. Additionally, there was also a main effect of target type (living/non-living), β = 0.01, SE = 0.004, *t* (47.68) = 3.08, *p* =.003, which is based on faster categorizations of living targets compared to non-living targets. Main effects were not qualified by any interactions, all *ps* > 0.14.

For the accuracy analysis (Table 2), it showed a significant main effect of category congruence (logit estimate = − 0.50, SE = 0.13, *p* <.001). However, there were no main effects of association (logit estimate = − 0.07, *p* =.217), or target type (logit estimate = 0.13, *p* =.229). There was also a significant interaction between category congruence and target type (logit estimate = − 0.15, SE = 0.06, *p* =.010), indicating that the effect of category match differed between living and non-living targets. None of the two-way interactions involving association reached significance, though the three-way interaction between association, category congruence, and target type showed a marginal trend (logit estimate = 0.10, SE = 0.06, *p* =.081). The follow-up test of the two-way interaction between category congruence and target type revealed that the categorical priming effect was stronger for non-living targets (*odds ratio = 0.28*, *z* = − 4.56, *p* <.001), compared to living targets (*odds ratio = 0.50*, *z* = − 2.60, *p* =.009). To further explore the three-way interaction, we examined the simple effects of association across combinations of category match and target type using estimated marginal means. A significant associative priming effect was observed only for non-living primes from the congruent category (*odds ratio = 0.555*, *z* = − 2.17, *p* =.030), but not for living primes from the congruent category (*odds ratio = 0.957*,* z* = − 0.197, *p* =.844).

**Table 2:** Means and standard deviations of accuracy rates (%) and response times (*ms*) as a function of prime-target relation

Target Type	Category Congruence	Association	Accuracy (%)		Response Times	
			*Mean*	*SD*	*Mean*	*SD*
Living	Incongruence	Associated	87.1	0.335	521	164
		Non-associated	87.5	0.331	538	207
	Congruence	Associated	94.9	0.221	511	207
		Non-associated	94.4	0.230	525	211
Non-living	Incongruence	Associated	86.2	0.345	549	228
		Non-associated	87.5	0.330	563	246
	Congruence	Associated	97.0	0.170	519	175
		Non-associated	95.4	0.209	540	204

### Diffusion modeling results

Graphical inspection of MCMC chains and $\:\widehat{R}$ values indicated good convergence of the algorithm across all seven models. Goodness-of-fit of models to the observed data was assessed with the LOOIC, with low values indicating good fit (Table 1). The baseline model (Model 0) that does not incorporate any priming effects showed the worst fit. Models 1 and 2 aimed at explaining associative priming by allowing differences in drift rate and non-decision time respectively. LOOIC values indicated a better fit for the latter model that incorporates different non-decision times for trial with associated vs. non-associated targets. Models 3 to 5 examined categorical priming by mapping it onto drift, non-decision time, or starting point, respectively. Results suggested that categorical priming is best explained by differences in drift rates (Model 3). Model 6 combined the models that accounted best for associative Priming (Model 2) and for category priming (Model 3) and thus allowed drift to vary between categorical match and non-match trials, whereas non-decision time is a function of association status. In Model 7 main effects but without interaction effects were included. In Model 8, main effects and an interaction for categorical and associative priming were allowed for both drift and non-decision time. Among all models tested, this model had the best fit. Finally, in the full model (Model 9), additionally an effect of prime category on relative starting point was included; however, this resulted in a deterioration of fit.

### Model specification

Here, we will describe in detail the specification of the best-fitting model (Model 8). In this model, drift rates and non-decision times for each trial were estimated as a function of prime-target relation. Specifically, we used effect coding for association status ($\:A\in\:\left[-\mathrm{1,1}\right]$, for non-associated vs. associated) and category congruence ($\:C\in\:[-\mathrm{1,1}]$, for non-match vs. match). We included random intercepts and random slopes across individuals for both predictors. Equation (1a, 1b) and (2) provide details how drift and non-decision time, respectively, of person $\:i$ in trial $\:j$ were calculated. Note that the priming effects on drift depend on target type, as associations and congruence is assumed to always push drift towards the correct response, that is, drift is increased for living targets (upper threshold) and decreased for non-living targets (lower threshold).

1a $$\:{v}_{living\left(ij\right)}={v}_{\left(i\right)}+{v}_{a\left(i\right)}\cdot\:{A}_{\left(j\right)}+{v}_{c\left(i\right)}\cdot\:{C}_{\:\left(j\right)}+{v}_{i\left(i\right)}\cdot\:{A}_{\left(j\right)}\cdot\:{C}_{\left(j\right)}$$

1b $$\:{v}_{non-living\left(ij\right)}={v}_{\left(i\right)}-{v}_{a\left(i\right)}\cdot\:{A}_{\left(j\right)}-{v}_{c\left(i\right)}\cdot\:{C}_{\:\left(j\right)}-{v}_{i\left(i\right)}\cdot\:{A}_{\left(j\right)}\cdot\:{C}_{\left(j\right)}$$

2 $$\:{t}_{00\left(ij\right)}={t}_{0\left(i\right)}+{t}_{0a\left(i\right)}\cdot\:{A}_{\left(j\right)}+{t}_{0c\left(i\right)}\cdot\:{C}_{\:\left(j\right)}+{t}_{0i\left(i\right)}\cdot\:{A}_{\left(j\right)}\cdot\:{C}_{\left(j\right)}$$

In these equations, $\:{v}_{a}$ and $\:{t}_{0a}$ are the effects of association, $\:{v}_{c}$ and $\:{t}_{0c}$ are the effects of category match, and $\:{v}_{i}$ and $\:{t}_{0i}$ are the corresponding interaction effects on drift and non-decision time, respectively.

### Parameter values

$\:\widehat{R}$ values for all parameters of Model 8 were below 1.01, effective sample sizes (ESSs) were larger than 400, and no divergence transitions were observed (Vehtari et al., 2021). Posterior predictive checks (see Supplemental material E) showed good fit of the predicted and observed data.

Posterior means for all fixed-effect parameters of Model 8 are displayed in Table 3. Values for the four basic diffusion model parameters lie within the range typically found in other studies (Tran et al., 2020). Random effects indicating individual differences are illustrated in Table S2 in Supplementary Material F. Especially for the category priming effect on drift rates, a large variability across subjects was observed.

**Table 3:** Means, standard deviations, and 95% credibility intervals of posterior distributions of the fixed effects in model 8

Parameters	Mean	SD	Lower bound	Upper bound
$\:{\mu\:}_{a}$	1.398	0.056	1.287	1.505
$\:{\mu\:}_{w}$	0.514	0.008	0.497	0.530
$\:{\mu\:}_{v1}$	−2.909	0.238	−3.361	−2.432
$\:{\mu\:}_{v2}$	3.030	0.243	2.542	3.484
$\:{\mu\:}_{t0}$	0.296	0.011	0.273	0.316
$\:{\mu\:}_{va}$	0.049	0.029	−0.005	0.107
$\:{\mu\:}_{vc}$	0.264	0.072	0.117	0.400
$\:{\mu\:}_{vi}$	−0.018	0.029	−0.077	0.037
$\:{\mu\:}_{t0a}$	−0.003	0.001	−0.006	−0.001
$\:{\mu\:}_{t0c}$	0.000	0.001	−0.003	0.002
$\:{\mu\:}_{t0i}$	−0.003	0.001	−0.005	0.000

To explain associative priming and category congruence effects, we are primarily interested in the effects of the prime-target relation on drift rates and non-decision times. There was only marginal evidence for a main effect of association on drift rates (mean $\:{v}_{a}$= 0.049; $\:95\mathrm{\%}\:CI=[-0.005,\:0.107]$), suggesting slightly faster evidence accumulation after associated primes. In contrast, the effect of category congruence on drift rates was much more pronounced (mean $\:{v}_{c}=$0.264; 95% $\:CI=[0.117,\:0.400]$). Results indicated no substantial interaction of both types of priming (mean $\:{v}_{i}=0.018$; $\:95\mathrm{\%}\:CI=[-0.077,\:0.037]$).

**Fig. 3:**
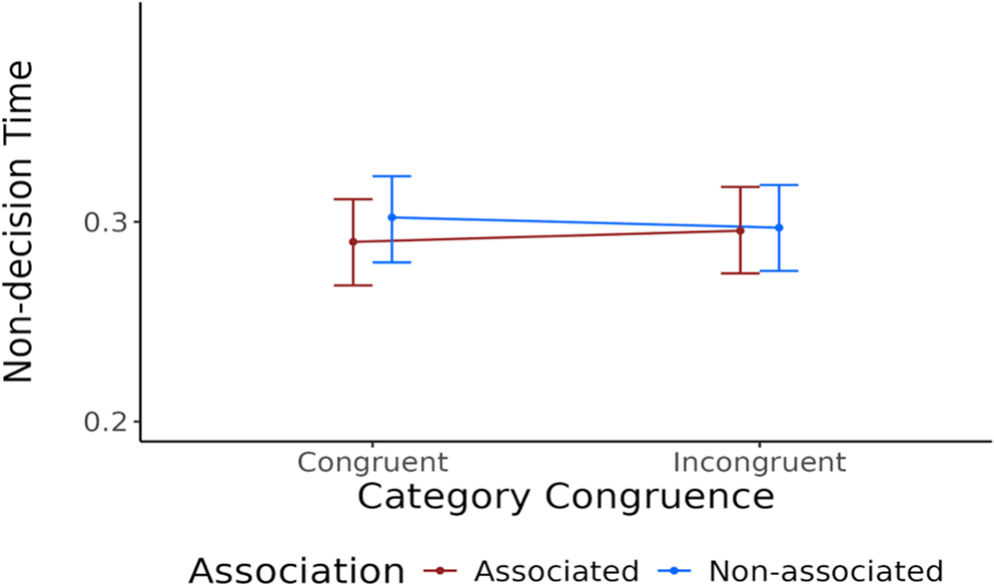
Non-decision time as a function of category congruence and semantic association. *Notes*: Points show the means; error bars show 95% credible intervals

Non-decision times were slightly reduced for trials with associated primes (mean $\:{{t}_{0}}_{a}=-0.003$, with $\:95\mathrm{\%}\:CI=[-0.006,\:-0.001]$), indicating a modest facilitation in early processing stages. In contrast, category congruence did not appear to affect non-decision time (mean $\:{{t}_{0}}_{c}=0$; $\:95\mathrm{\%}\:CI=[-0.003,\:0.002]$). An interaction between association and category congruence indicates a strongest decrease of non-decision time in trials featuring associated primes from the same category (mean $\:{{t}_{0}}_{i}=-0.003$; $\:95\mathrm{\%}\:CI=[-0.005,\:0.000]$; see Fig. 3).

## Discussion

In the following, we will first outline that, although the pattern from DM analysis may appear partly inconsistent with previous findings at first glance, possible explanations can be found if we take into account differences between word processing and picture processing. It has been observed before – and in fact is one of the core assumptions of drift diffusion modeling in priming research – that priming is a phenomenon that can affect different processes in different ways. However, the observed differences between picture and word targets, and between associative and categorical priming should be explainable in a common framework. In the second part of the discussion, we will outline ideas on how modality-specific characteristics of conceptual activation, together with the specific properties of association and category congruence, contribute to the relative strength and locus of priming effects.

An associational relationship reflects co-occurrence patterns and transition probabilities, that is, the embedding of an object in a textual or real-world context. This has been linked to predictive processing, understood as pre-activation of related (in the original account holistic) ‘nodes’ such as lexical entries or concepts via spreading activation (see, e.g., Mech et al., 2022). In contrast, feature overlap links an item to other items that share similar properties, thereby forming a category. This facilitates access to semantic knowledge for that category. More in detail, we will propose that words and pictures differ in how and when they access semantic knowledge and, in how they can utilize the cues provided by the prime words. These differences give rise to distinct patterns in diffusion model parameters for associative and categorical priming across word and picture targets.

### Effects of associative priming on the diffusion model parameters

Our modelling results revealed that the processing of associated primes accelerated non-decision processes in target classification: Goodness-of-fit was better for a model that mapped prime-target associations on non-decision times compared to models that allowed for different drift rates. Also, the posterior distributions from the final model showed a robust effect of prime-target associations only for non-decision time, whereas the impact on drift rate was less pronounced. This suggests that association did not or hardly facilitate evidence accumulation in our experiment.

This finding, on the surface, is not consistent with previous results (Gomez et al., 2013; Voss et al., 2013b). Voss and colleagueVoss et al. (2013a, b) showed that—in a word-word priming paradigm—associated primes increased drift rates in both lexical decision and semantic categorization. They argued that this reflects facilitated target processing, as those target concepts were pre-activated through spreading activation from the prime concepts, making evidence accumulation more efficient^8^. As described in the Introduction, if semantic priming occurs predominantly at a conceptual level, and if concepts are shared across modalities, then we should observe the same general pattern with word primes and picture targets regarding evidence accumulation.

In our study, the effect of prime-target associations on drift rate was only marginal. Assuming that the key difference from previous studies lies in the modality of the target, a possible explanation is that the priming effects found in previous studies relied largely on language-specific processing stages such as lexical access. In addition, the change in target modality could have modulated priming mechanisms at the conceptual level. We will now explain these two options in more detail. In contrast to our findings, Todorova and Neville (2020) observed in their word-picture priming study an effect of associations on drift, suggesting a facilitation effect of related/congruent primes compared to neutral primes and an interference effect of unrelated/incongruent compared to neutral primes. We will discuss their findings in the context of the framework below.

One possible explanation is that the pre-activation of the target concept in previous studies occurred primarily at lexical access. The presence of priming effects in the semantic categorization task might suggest that associated primes not only help access targets in the lexicon, but also access their semantic representations. However, it is still possible that the facilitated target classification was mediated by the pre-activation at a lexical level. In contrast, the present task required the categorization of pictures, which is unlikely to depend on access to lexical representations. This could explain why we did not observe any clear effect of association on drift with picture targets. Supporting this idea, Taikh et al. (2015) showed that reaction times in the classification of word targets were mainly influenced by lexical as opposed to semantic factors.

Another possible reason for the absence of a strong effect of associations on drift in our study is the assumption that semantic associations differ for pictures and words. The selection of prime stimuli in our study was based on lexical-level association strength. In the database we used (De Deyne et al., 2019), association strength is calculated based on the first three words participants spontaneously generated in response to a cue word. Therefore, the association of prime and target in the experiment is based on the lexical level rather than on the word-picture association. Saffran et al. (2003) used words and corresponding pictures as stimuli and asked participants to report the first word that came to their mind. The results showed that words tended to evoke common verbal associations (e.g. lion-tiger, tractor-trailer, bride-groom), whereas pictures elicited often associations related to perceptual features (e.g., tree-shadow; corn-yellow). This might suggest that words and pictures may activate different representations that stress different features (see also Truman & Kutas, 2024). Even though the primes in our study were words, the modality-dependent nature of associative activation could still have played a role. It is possible that the triggered lexical activation by the associated prime actually did not closely relate to the picture target. As a result, this could have diminished the effect of association on evidence accumulation. For future research using cross-modal prime-target pairs, it would be advisable to re-measure the strength of the associations.

The two possible explanations, that previous effects relied primarily on modality-specific mechanisms like lexical access, and that word-derived association norms are less effective for picture targets than for word targets, are not mutually exclusive. Both explanations could contribute to the reduced or absent association effect on drift.

On the other hand, there was an effect of association on non-decision time in our study, which speaks against associations only working at a lexical level: Associated prime words *do* affect subsequent picture target processing. Interestingly, this effect would not be predicted by findings from word-word priming literature, where association effects on non-decision time were inconsistent and largely limited to lexical decision tasks. The explanations from previous literature, that this may reflect a faster orthographical analysis or a retrospective semantic matching process, only apply to lexical decision tasks. Therefore, these mechanisms do not provide any plausible explanation for our results. Taken together, our findings indicate that the associative effect is not simply weaker or absent, but has changed in nature. It no longer (or to a much lesser degree) affects drift rate, but instead emerges at a non-decisional processing stage.

Non-decision time is an aggregate of the duration of pre-decisional (commonly interpreted as encoding) and post-decisional (commonly interpreted as motor response planning and execution) processes. In our experimental design, an effect of association on response execution stage denoted by non-decision time seems implausible. In the lexical decision task, where effects of association on non-decision time mostly have been observed, association is coupled with a specific response (“yes, it is a word”), so that an effect at the response level is conceivable (Klauer & Musch, 2002). In our word-picture paradigm with a categorization task, however, association is orthogonal to the required response in the balance design, so that association is not systematically linked to the response. Therefore, response-related mechanisms are unlikely to cause the observed effect on non-decision time. We hence suggest that the observed effect on non-decision time reflects an advantage in visual encoding for associated primes. Further arguments for this view will be discussed below in the section ‘Associative Priming in the Word-Picture Paradigm’.

### Effects of categorical priming on the diffusion model parameters

In the present study, we employed a semantic categorization task, in which—in contrast to naming or lexical decisions—semantic relatedness is aligned with response congruence. Specifically, category-matched prime-target pairs share the semantic feature of being living or not, which is the feature that needs to be selected for the (semantic) decision. Semantic category congruence could affect decisional processes, mirrored on drift rate in a DM framework. This is consistent with the ‘task-set perspective’, which assumes that primes could pre-activate the associated response code at the abstract level (Logan & Gordon, 2001; Vandierendonck et al., 2010), thereby influencing the decisional process.

In our experiment, we found effects of category match of primes and targets for both RTs and accuracy. The DM analysis revealed that these effects were based on differences in drift rates, but not non-decision times, suggesting a facilitation/competition account at the decision selection stage rather than the response preparation and execution at the motor stage. Model selection furthermore suggested no influence of category congruence on the relative starting point. As opposed to our results, in the word-word priming studies by Voss et al. (2013b) categorical priming exclusively mapped on non-decision times and had no impact on drift.

We proposed in our hypotheses that categorical priming effects on drift rate are theoretically plausible. The results support the semantic account of categorical priming. A review by Hutchison (2003) on purely associative vs. purely semantic priming concludes that “pure” semantic priming with little feature overlap (corresponding to our congruent, non-associated condition) does not seem to produce reliable automatic priming effects (see Lucas, 2000, for a different conclusion). This may be true if semantic priming is seen as a mechanism purely driven by the presumably stable connections between concepts in semantic memory. However, both Hutchison (2003) and Lucas (2000) excluded semantic categorization tasks with feature overlap in their reviews on purely semantic priming, arguing that the effect in that case could be driven by purely response-based priming in the absence of pure semantic priming. We would thus argue that our semantic categorization task produced a task-induced form of semantic priming, as feature overlap is relevant to the task at hand and thus forms part of the task-set. Specifically, a prime might activate the task-relevant category (e.g., living/non-living) and thereby facilitate recognizing the target in the same category. Conversely, the pre-activation of an opposing category could introduce interference, thereby slowing the decisional process. Diffusion model analysis helped us distinguish that the locus of task-induced response priming was not at the motor stage, but at the decisional stage.

### Towards a shared framework

So far, we have related our results to the existing literature of diffusion modelling on word-word priming, but our findings can be understood more comprehensively in a modality-specific concept activation framework that explains the different findings by relating them to differences in concept activation in the processing of words and pictures.

#### Word processing

The processing of written words follows a pathway for symbol processing. First, a word must be visually recognized and located within the mental lexicon, before its conceptual meaning can be accessed. As the visual form of a word is not inherently tied to its meaning (e.g., ‘look’ and ‘lock’ are orthographically and phonological similar words, but they are very different in meaning), the identification of orthographic form is essential before lexical and subsequent semantic access. In diffusion modelling, the time required to achieve this quality of visual representation is captured in the encoding part of non-decision time (Ratcliff & Smith, 2010).

Lexical access can be aided by associative relations. Language comprehension usually occurs under time pressure and with temporarily ambiguous input, so the brain relies on entrenched co-activation patterns to aid retrieval. These patterns often reflect high-frequency co-occurrences in language use and provide a powerful predictive mechanism. Recent approaches are therefore able to measure associative relations statistically rather than through human ratings (Hofmann et al., 2018; Roelke et al., 2018).

Semantic access should be differentiated from lexical access, yet it can also profit from an associated prime for the same reason, i.e., facilitated retrieval due to frequent co-occurrence. Lexical access occurs first, followed by semantic access, which unfolds conceptual information linked to the lexical entry^9^. While older approaches tended to view concepts as the stable meaning of a word (whether holistic or compositional), the newer literature points out that feature activation is dynamical, graded, and guided by contextual cues (e.g., Truman & Kutas, 2024; Lupyan & Thompson-Schill, 2012). Accordingly, a prime can facilitate and modulate the activation of a target’s semantic features by providing a compatible conceptual context. This highlights the facilitating role of common co-occurrence patterns at the conceptual in addition to the lexical level.

In drift diffusion modelling, both lexical and semantic access should contribute to evidence accumulation, to the extent that they are necessary for extracting task-relevant information. Consistent with this view, associative priming has been found to speed up drift rate in lexical decision task (where semantic access is optional) and semantic categorization (where semantic access is essential). However, we did not find the same strong effect of association on drift for picture targets. We will return to this point below, after contrasting the processes of conceptual activation in pictures.

#### Picture processing

Picture processing offers a more direct pathway to semantic activation, as visual features inherently carry meaning in a more analog manner. For images, similarity in visual input usually correlates with similarity in meaning. For example, a wolf and a dog share numerous visual features and belong to related categories. In early visual processing, features such as shape, color, and texture are extracted from the input. Unlike in word processing, where features must be retrieved from long-term memory via a lexical entry, visual features here are the primary information used to access conceptual representations in long-term memory. These conceptual representations can in turn feed back on visual processing in a highly interactive manner, as well as serve to retrieve further semantic knowledge.

Notably, whereas words by virtue of their nature link their referents to a specific category (in the sense that words are category labels), pictures always show specific, concrete instances. A single image may show a small brown dog, furry, sitting with a sleepy expression on his face, seen from the front. If we view it as an instantiation of a concept, it is open to being linked to concepts at very different levels of the taxonomic hierarchy (e.g., *poodle*, *dog*, *animal*), or even categories based on different types of commonalities (e.g., *something cuddly*). The choice is modulated by context and task demands.

In the time course of visuo-semantic picture processing, meaning representation and visual representation develop jointly and interactively from coarse to fine-grained over time (e.g., animal > dog > poodle). That means, it is possible to recognize an image depicting an animal before identifying it as a dog and more specifically a poodle, even if the whole process is extremely fast (Clarke et al., 2011; Fabre-Thorpe, 2011). Conceptual information thus becomes available earlier than in word processing, a phenomenon often referred to as the “picture superiority effect” (Irwin & Lupker, 1983; Potter & Faulconer, 1975).

In the context of diffusion modelling, this raises the question of when encoding finishes and evidence accumulation starts. Ratcliff and Smith (2010) discussed how the onset of evidence accumulation was related to visual processing and suggested that it depended on the quality and stability of the stimulus representation. What they deem a biologically plausible possibility is that there might be an initial inhibition mechanism that first needs to be released once the representation achieves a certain threshold quality. In the case of word processing, this representation then serves as the foundation for accessing the lexicon. By contrast, we propose that in picture processing this early representation possesses at least a gist-like quality and already carries meaning. The “gist” refers to the main idea or essence of what the picture depicts: a visually stable yet still coarse representation that provides a first, best guess about the image’s content and serves as a basis for more detailed exploration. This proposal is consistent with what is known about picture processing and the variation and length of nondecision times ($\:{t}_{0}$) in the diffusion model.

Support for the separability of encoding and decision processes and a proposal on how to distinguish between them comes from a study by Nunez et al. (2019). The authors linked electrophysiological recordings to DM parameters and found that the onset of the N200 (a negative going peak at around 200 ms after stimulus onset) was associated with the length of non-decision time, and thus potentially marks the transition from pre-decisional to decisional processes (for a similar estimate of the beginning of decisional processes, see VanRullen & Thorpe, 2001; though Bompas et al., 2024, proposed a much shorter pre-decisional time).

Interestingly, these findings suggest that neuronal activation patterns distinguish animacy even before the onset of decisional processes. For example, different activation distinguishing animals from objects based on visual differences occurred as early as 75 ms after stimulus onset in EEG measures (VanRullen & Thorpe, 2001). However, this early category differentiation in picture processing does not necessarily imply awareness, which is believed to arise around 100–150 ms after stimulus onset, when the fast feedforward sweep of the signal reaches higher cortical areas and allows for the recognition of the gist of the image (Hochstein & Ahissar, 2002; Lamme & Roelfsema, 2000). By about 180 ms after stimulus onset, neurophysiological responses already show differences that cannot be explained by visual features alone, suggesting that conceptual information is being processed (Giari et al., 2020). Thus, decisional processes in picture processing operate on a representation that already contains categorical information, but this still needs to be refined and linked to the appropriate response.

One important question is whether these early visual processes could in principle be primed by a word cue. In fact, there is growing evidence that the encoding stage in picture processing can be influenced by linguistic context, such as a preceding word prime. Over the past two decades, various reviews have demonstrated that early visual processing can be influenced by top-down influences, such as expectations and language-based cues (Simanova et al., 2016; Collins & Olson, 2014; Lupyan et al., 2020). Our results indicating an effect of association on non-decision time contribute to the growing body of evidence.

### Associative priming in the word-picture paradigm

We propose that the modality differences explain the divergent patterns observed in word-word and word-picture priming studies. In prior word–word priming studies, associative priming typically modulated drift rates, which is consistent with the notion that associations support lexical and semantic access. In our word-picture paradigm, however, we found that prime-target association sped up the non-decision time instead. This is, an associated prime word (e.g., “violinist” preceding a picture of a violin) did not significantly accelerate evidence accumulation, but it slightly reduced the overall processing time outside the decision phase (by about 6 ms).

This shift in the locus of priming suggests that the same underlying mechanism – spreading activation or contextual prediction – was engaged in both modalities but impacted different processing stages. For word targets, associated concepts facilitate prediction of upcoming lexical items and/or semantic features, thereby speeding up evidence accumulation. For picture targets, association are likely to operate earlier, during encoding, by attuning the visual system to anticipate specific features or helping to construct the initial gist representation by providing a better and faster ‘first guess’ of the meaning. This accelerates the construction of coherent object representation, effectively speeding up the onset of the decision process. Additionally, the effect of association on non-decision time was enlarged by an interaction with congruency: associated and congruent primes often shared more visual features (e.g., cello – violin), suggesting that associative priming may involve early visual attunement to target shape (Gilbert & Li, 2013).

As discussed earlier, the effect of associative priming on non-decision time likely reflects a modulation in pre-decisional processes (visual ‘encoding’), not post-decisional processes, taken to comprise motor planning and execution. However, mapping psychological processes onto the non-decision component has to be taken with caution, because diffusion model treats all pre- and post-decisional operations as a single residual term. To address this limitation, future research could employ alternative sequential-sampling models that explicitly represent early perceptual dynamics. One promising approach is the two-stage diffusion framework (Diederich & Busemeyer, 2006; Diederich, 2016, 2024; Diederich & Colonius, 2021). Within this framework, the decision process is assumed to unfold in two successive stochastic stages, each characterized by distinct evidence accumulation speeds (i.e. drift rates). In the context of a priming task, the first stage can be conceptualized as a stochastic process that begins with the prime presentation. Upon target presentation, the system transitions to the second stage, accumulating evidence related to the target stimulus towards one of the decision boundaries. Future work could test whether this two-stage diffusion framework better captures priming with picture targets, where meaning begins to emerge during early visual encoding. This approach treats the influence of prime as an early decision-like process, so this framework may offer a more mechanistic account of how associative activation unfolds over time, naturally distinguishing between perceptual, decisional, and motor execution processes.

### Categorical priming in word-picture paradigm

The cross-modal configuration between prime and target is also likely to highlight the semantic processes involved in categorical priming. We observed that category congruent primes enhanced drift rate, suggesting conceptual facilitation: Word primes may pre-activate the picture target’s category, induced by the task-set, thereby facilitating extraction of the task-relevant feature (e.g., living or non-living) during evidence accumulation. Picture targets may be more susceptible to categorical priming than word targets, as pictures offer richer perceptual input than words, enabling flexible links to conceptual representations at multiple levels or foci (e.g., animal, dog, poodle, or something cuddly, etc.).

We found no interaction between association and category congruency in behavioral results or drift rates: Prime-target pairs that were both associated *and* congruent did not show stronger priming than associated alone. The simple congruence of animacy (animate vs. inanimate) appeared sufficient to facilitate processing. This pattern parallels Todorova and Neville (2020), who found effects on drift rate and non-decision time for both associated and category-congruent primes as well as for identity primes, yet identity primes with greater feature overlap did not yield stronger effects. The fact that only the overlap in the task-relevant feature matters, rather than the strength of semantic relatedness, suggests different processes are involved. Todorova and Neville interpreted their finding as evidence of experienced processing fluency rather than spreading activation: a metacognitive sense of ease that guides decisions (cf. also Potter et al., 2018). Notably, such an experience of fluency likely arises from semantic-level pre-activation (Alter & Oppenheimer, 2009), which is consistent with the idea that congruent primes in our study facilitated conceptual rather than motor processes.

From a task-set perspective, task rules can operate across modalities and may pre-activate the category-relevant response code at an abstract level, without directly engaging in motor preparation and execution reflected by the non-decision time. While this view aligns with our cross-modal findings of categorical priming, it is inconsistent with the results of Voss et al. (2013b). In their study (Experiment 2) which was designed to avoid action-trigger and S-R mapping, category-match effects were observed in non-decision time. One possible explanation is that the effect on non-decision time observed in their study may not reflect pure motor-stage processing, but rather post-decisional control and monitoring processes. Recent work has challenged the classical view that motor response execution can be entirely separated from decisional stage in evidence accumulation models. For example, Servant et al. (2021) and Weindel et al. (2021), using perceptual decision tasks, have demonstrated that evidence accumulation continues even after a decision has been made. Similarly, Scaltritti et al. (2023) also falsified the classical assumption using conceptual decision tasks. They provided a functional explanation of this post-decisional stage reflecting a verification and/or monitoring mechanism. Once the initial decision threshold has been reached, continued accumulation represents a second-order decision variable that is used for confidence evaluation and error monitoring (Desender et al., 2021). The unfolding of meaning for words depends more heavily on context, whereas picture meaning is more directly anchored in perceptual input. Consequently, verification or monitoring processes may play a greater role in the processing of words than for pictures, where conceptual meaning is more immediately available.

#### Dynamic processes of the priming effects

Another observation that supports the different nature of semantic and associative priming is their differential modulation by learning effects. Because targets were repeated four times preceded by different primes, we examined how priming effects changed across trials using an effect course analysis (Berger et al., 2024), which enables a fine-grained, continuous examination of the dynamic effects across trials for both associative priming and categorical priming (see Supplementary material G). Associative RT priming became only reliable, i.e. associated with a significant cluster, after more than a third of the experiment, whereas categorical RT priming could only be observed at the beginning of the experiment, reflected by a significant cluster, but decreased with practice. We interpret the decline in categorical priming effect as the result of stimulus-response associations forming for the targets during the experiment. This gradually evolving short-cut would override the effect of congruency priming during the experiment. On the contrary, the effect of association gained stability after the first third of the experiment, suggesting that it may reflect attunement of anticipatory processes functioning like priors on visual features or gist.

## Conclusion

In sum, we propose that both priming mechanisms can in principle occur in the cross-modal paradigm: Associative priming works by enriching the context and supporting the generation of features (words) or the integration of features (pictures) needed to build the representation suitable for a decision. Category congruency facilitates the identification and abstraction of task-relevant features from its decisional context, especially when the input is rich (pictures). Response congruence, while potentially providing a short-cut or head-start for motor processes in unimodal tasks, did not transfer to this level in our cross-modal setting, confirming earlier observations in the literature. In short, the relative timing and impact of priming effects depend on the use that modality-specific and task-dependent processing patterns can make of the cues provided by the prime.

## Supplementary Information

Below is the link to the electronic supplementary material.

Supplementary Material 1 (DOCX 6.90 MB)

## Data Availability

The raw data and code of the present study are available via the Open Science Framework (OSF) and can be accessed at https://osf.io/h8ceg/.

## Footnotes

1 In this study, the term cross-modal specifically refers to the interaction between verbal and pictorial representations. Although both prime and target stimuli were presented visually, they belong to different representational modalities (i.e., language and imagery), rather than to different sensory modalities such as vision and audition.

2 Both models offer explanations for semantic priming effects based on associative relationships and semantic similarity. While the spreading activation model primarily accounts for associative links, it also includes possible paths between nodes to explain feature-based similarity. Conversely, the distributed memory model primarily explains feature overlap, but can also account for learned associations.

3 The mechanism of category congruence priming has been debated especially in the context of subliminal priming (for a review, see Kouider & Dehaene, 2007; cf. also Van den Bussche et al., 2009b).

4 The amount of inherent noise in the evidence accumulation process is controlled by the diffusion constant, which serves as a scaling parameter that does not change the relationships between parameters in DM. In this study, a diffusion constant of *s* = 1 was used.

5 Estimating the frequency of a picture stimulus (e.g., a picture of a bear) from the corresponding word (“bear”) makes sense, when you assume that both share the same semantic representation (i.e., an object concept; cf. Gregorova et al., 2023).

6 In diffusion modelling, fast outliers are especially harmful (Lerche et al., 2017). Because of the positive skew of RT distributions, it is recommended to perform outlier detection based on logarithmized data, although then untransformed data are entered into the DM analyses.

7 Recently, the full diffusion including inter-trial variabilities has been implemented in Stan (Henrich et al., 2024). We first tried to estimate models including inter-trial variability of non-decision time ($\:s{t}_{0}$), which might help improve the robustness of a DM analysis (Boehm et al., 2018a, b; Lerche & Voss, 2016). However, models including interaction effects (Model 8 and 9) did not converge well when $\:s{t}_{0}$ was included as a free or fixed ($\:s{t}_{0}$=0.1) parameter. The model comparison results from Model 1 to Model 6 showed the same trend as the results presented here.

8 In distributed memory models, association usually is modeled as facilitated transition from one representational state to the other, which would equally lead to faster target processing.

9 As most concept theories–not only embodied ones–assume, apart from stored semantic knowledge concepts may include multimodal sensory information. Notably, this may also include visual and especially shape information of the named object (see, e.g., Lupyan & Thompson-Schill, 2012; Truman & Kutas, 2024). This will become relevant for picture targets, as will be argued below.
