# Supplementary material for: Associative and categorical priming in a word-picture paradigm: a diffusion model analysis
Source: Psychol Res. 2026 Feb 7;90(1):24. doi: 10.1007/s00426-025-02234-w (PMC12881110; doi:10.1007/s00426-025-02234-w)

# Supplementary Material

## Supplementary Material A: Stimuli

**Table S1**

| Prime word | Living Target | Description | Prime word | Non-living Target | Description |
| --- | --- | --- | --- | --- | --- |
| panda | 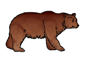 | bear | cigarette |  | ashtray |
| polar |  |  | smoker |  |  |
| rider |  |  | loaf |  |  |
| broth |  |  | rodent |  |  |
| caterpillar | 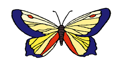 | butterfly | timber |  | axe |
| ﻿monarch |  |  | woodsman |  |  |
| termite |  |  | blade |  |  |
| cigarrette |  |  | baker |  |  |
| puppy |  | dog | pedal |  | bike |
| bark |  |  | rider |  |  |
| biker |  |  | cap |  |  |
| carrot |  |  | lion |  |  |
| kitty |  | cat | novel |  | book |
| whisker |  |  | reader |  |  |
| sniper |  |  | saddle |  |  |
| venom |  |  | primate |  |  |
| poultry |  | chicken | shuttle |  | bus |
| broth |  |  | driver |  |  |
| boa |  |  | aquarium |  |  |
| mop |  |  | mammoth |  |  |
| mammoth |  | elephant | loaf |  | bread |
| tusk |  |  | baker |  |  |
| barber |  |  | twinkle |  |  |
| instrument |  |  | insect |  |  |
| termite |  | ant | mop |  | broom |
| antenna |  |  | witch |  |  |
| butcher |  |  | bark |  |  |
| whisker |  |  | woodsman |  |  |
| santa |  | reindeer | tiara |  | crown |
| sleigh |  |  | king |  |  |
| puppy |  |  | shuttle |  |  |
| shears |  |  | musician |  |  |
| salmon |  | fish | prom |  | dress |
| aquarium |  |  | bride |  |  |
| tadpole |  |  | pistol |  |  |
| trap |  |  | bunny |  |  |
| tadpole |  | frog | motorbike |  | helmet |
| leap |  |  | biker |  |  |
| shepherd |  |  | polar |  |  |
| mittens |  |  | referee |  |  |
| pony |  | horse | lenses |  | glasses |
| saddle |  |  | scholar |  |  |
| pianist |  |  | origami |  |  |
| pouch |  |  | salmon |  |  |
| wallaby |  | kangaroo | mittens |  | gloves |
| pouch |  |  | boxer |  |  |
| tailor |  |  | tusk |  |  |
| tiara |  |  | poultry |  |  |
| lion | 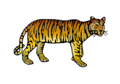 | tiger | bass |  | guitar |
| jungle |  |  | musician |  |  |
| witch |  |  | train |  |  |
| pedal |  |  | mollusk |  |  |
| primate |  | monkey | pistol |  | gun |
| banana |  |  | sniper |  |  |
| smoker |  |  | novel |  |  |
| timber |  |  | wallaby |  |  |
| rodent |  | mouse | braid |  | hair |
| trap |  |  | barber |  |  |
| ballerina |  |  | antenna |  |  |
| jungle |  |  | king |  |  |
| bunny |  | rabbit | cap |  | hat |
| carrot |  |  | cowboy |  |  |
| chipmunk |  |  | shell |  |  |
| braid |  |  | reader |  |  |
| shepherd |  | sheep | blade | 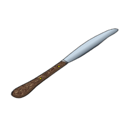 | knife |
| wool |  |  | butcher |  |  |
| kitty |  |  | cello |  |  |
| web |  |  | driver |  |  |
| mollusk |  | snail | instrument |  | piano |
| shell |  |  | pianist |  |  |
| celebrity |  |  | lenses |  |  |
| nuts |  |  | caterpillar |  |  |
| ballerina |  | swan | shears |  | scissors |
| origami |  |  | tailor |  |  |
| violinist |  |  | ﻿monarch |  |  |
| leap |  |  | boxer |  |  |
| boa |  | snake | cello |  | violin |
| venom |  |  | violinist |  |  |
| scholar |  |  | banana |  |  |
| motorbike |  |  | cowboy |  |  |

*Note.* The order of the prime words for each target picture is associated and congruent category, associated and incongruent category, non-associated and congruent category, and non-associated and incongruent category.

## Supplementary Material B: Comparison of Fixed-Effect Estimates Between Maximal and Final Models

**Table S2 *Fixed-Effect Estimates from Full and Final LMM for RT Analysis***

|  | Full Model | | | | Final Model | | | |
| --- | --- | --- | --- | --- | --- | --- | --- | --- |
|  | *b* | *SE* | *t* | *p* | *b* | *SE* | *t* | *p* |
| Intercept | 0.531 | 0.022 | 24.592 | <.001*** | 0.531 | 0.021 | 24.821 | <.001*** |
| A (Association) | 0.009 | 0.003 | 3.113 | 0.003** | 0.009 | 0.003 | 3.274 | 0.003** |
| C (Category  Congruence) | 0.008 | 0.003 | 2.343 | 0.024* | 0.008 | 0.003 | 2.673 | 0.011* |
| T (Target Type) | 0.011 | 0.004 | 2.829 | 0.007** | 0.012 | 0.004 | 3.079 | 0.003** |
| A × C | 0.000 | 0.002 | 0.039 | 0.969 | 0.000 | 0.002 | 0.030 | 0.976 |
| A × T | 0.001 | 0.002 | 0.233 | 0.817 | 0.000 | 0.002 | -0.030 | 0.976 |
| C × T | 0.003 | 0.003 | 1.088 | 0.282 | 0.004 | 0.003 | 1.514 | 0.140 |
| A × C × T | 0.000 | 0.002 | 0.073 | 0.942 | 0.000 | 0.002 | -0.123 | 0.902 |

*Note*. ****p* < .001, ***p* < .01, **p* < .05

**Table S3 *Fixed-Effect Estimates from Maximal and Final GLMM for Accuracy Analysis***

|  | Full Model | | | | Final Model | | | |
| --- | --- | --- | --- | --- | --- | --- | --- | --- |
|  | *b* | *SE* | *z* | *p* | *b* | *SE* | *z* | *p* |
| Intercept | 3.410 | 0.273 | 12.496 | <.001*** | 3.333 | 0.263 | 12.653 | <.001*** |
| A(Association) | -0.070 | 0.065 | -1.079 | 0.281 | -0.070 | 0.056 | -1.234 | 0.217 |
| C (Category  Congruence) | -0.508 | 0.137 | -3.712 | <.001*** | -0.498 | 0.126 | -3.949 | <.001*** |
| T(Target Type) | 0.080 | 0.122 | 0.651 | 0.515 | 0.125 | 0.104 | 1.204 | 0.229 |
| A × C | 0.084 | 0.061 | 1.391 | 0.164 | 0.089 | 0.056 | 1.572 | 0.116 |
| A × T | -0.027 | 0.066 | -0.409 | 0.683 | -0.038 | 0.056 | -0.668 | 0.504 |
| C × T | -0.134 | 0.067 | -1.979 | 0.048* | -0.146 | 0.057 | -2.586 | 0.010** |
| A × C × T | 0.097 | 0.069 | 1.412 | 0.158 | 0.099 | 0.056 | 1.746 | 0.081 |

*Note*. Coefficients (*b*) are on the logit scale (i.e., log-odds). ****p* < .001, ***p* < .01, **p* < .05

## Supplementary Material C:

In the second LMM target repetition (1 to 4) was included as an additional predictor using sum contrast coding. This analysis revealed that RTs significantly decreased with increasing target repetitions, *F* (3,60.55) = 10.01, *p* <.001. Inclusion of this predictor did not change core patterns of results, with *F* (1, 30.01) = 9.86, *p*=.004, for association status, F (1, 40.77) = 7.10, *p* =.011, for category congruence, and F (1, 48.49) = 7.71, p = .008 for target type.

There was no interaction between association status and target repetition, F<1. In contrast, the interaction between category congruence and target repetition approached significance, F (3, 5797.16) = 2.29, p = .076, indicating that the category congruence effect diminishes with increasing target repetition. A similar trend was found for the three-way interaction of category congruence, target type, and target repetition, F (3, 5739.13) = 2.35, p = .070, indicating potential modulation of category priming by both target type and repetition. No other higher-order interactions reached significance (ps > .70).

To follow up on the trend-level interaction between category congruence and target repetition, we conducted pairwise comparisons of the category condition at each level of target repetition (Figure S1). The follow-up tests indicated that the category congruence priming effect was significant when targets were shown the first time (z = 3.79, p = .0006), whereas there was no significant category congruence priming effect when they were repeated from the second to fourth time (all *p*>.48). These p-values were adjusted using the Holm correction for multiple comparisons.

Figure S1 Correct RTs as a function of Category Congruence and Target Repetitions.


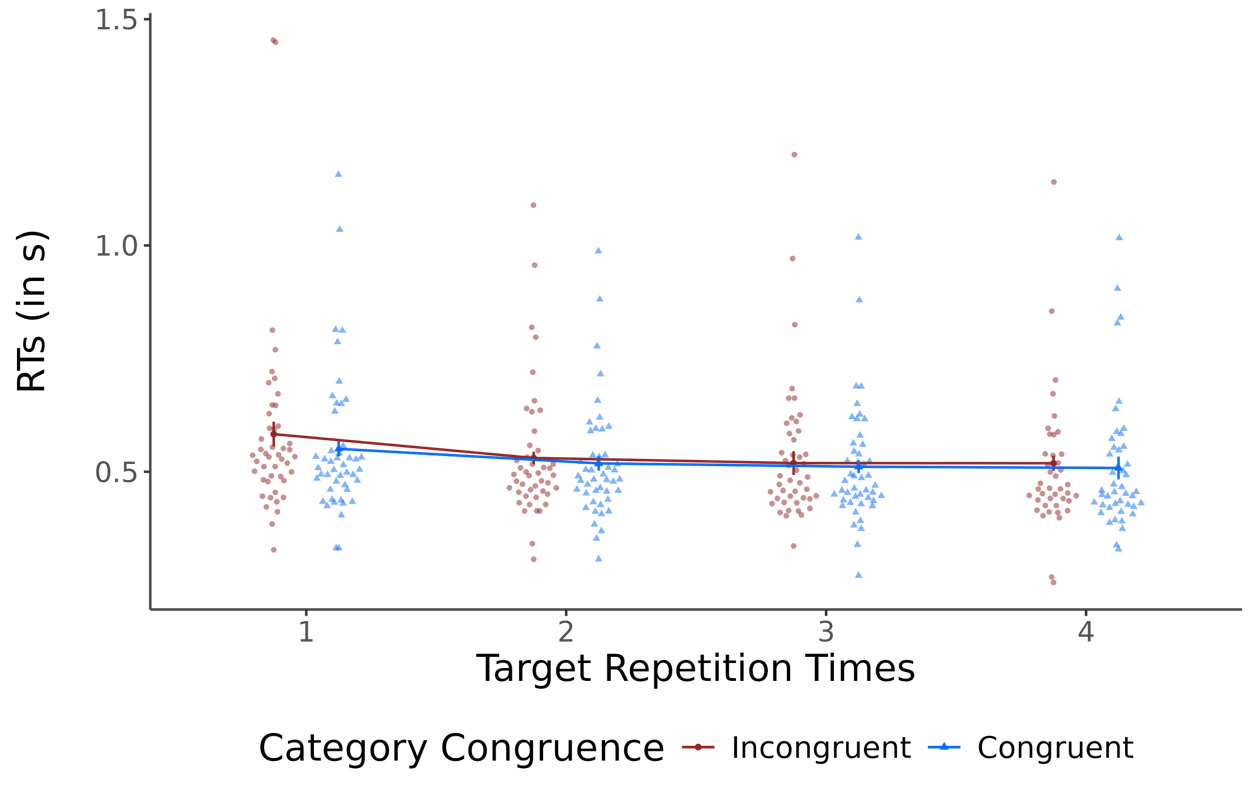


*Notes*: Points show individual participant means, lines show values averaged across participants, and error bars denote 95% within-subjects confidence intervals of the means.

## Supplementary Material D: Prior specifications in Model 8

For the association and category congruence effects on drift rates and non-decision times we used standard normal priors:

$$\mu_{\mathrm{va}} , \mu_{\mathrm{vc}} , \mu_{\mathrm{vi}} \sim normal(0, 0.5)$$

$$\mu_{t0a} , \mu_{t0c} , \mu_{t0i} \sim normal(0, 0.5)$$

The weakly informative priors for fixed effects of main parameters in drift diffusion model was chosen:

$$\mu_{a} \sim normal^{+}\left( 1, 2 \right)$$

$$\mu_{w}\sim beta\left( 5, 5 \right)$$

$$\mu_{v} \sim normal \left( 0, 5 \right)$$

$$\mu_{t0}\sim normal^{+} \left( 0.3, 0.2 \right)$$

The priors of standard normal with mean 0.3 and standard deviation of 1 is often chosen, but this wide prior led to convergence issue in model 8. Thus, we changed a narrower prior with the same mean value. The priors for threshold and non-decision were constrained to positive, which in line with diffusion model parameters’ range.

Priors for the random effects were chosen as following values:

$$\sigma_{a} , \sigma_{t0} , \sigma_{va}, \sigma_{vc}, \sigma_{vi}, \sigma_{t0a}, \sigma_{t0c} , \sigma_{t0i} \sim normal^{+}\left( 0. 0.2 \right)$$

$$\sigma_{w} \sim normal^{+}\left( 0, 0.1 \right)$$

$$\sigma_{v} \sim normal^{+}\left( 0, 2 \right)$$

## Supplementary Material E: Posterior Predictive check

**Figure S2 Posterior predictive check (1) for model 8**


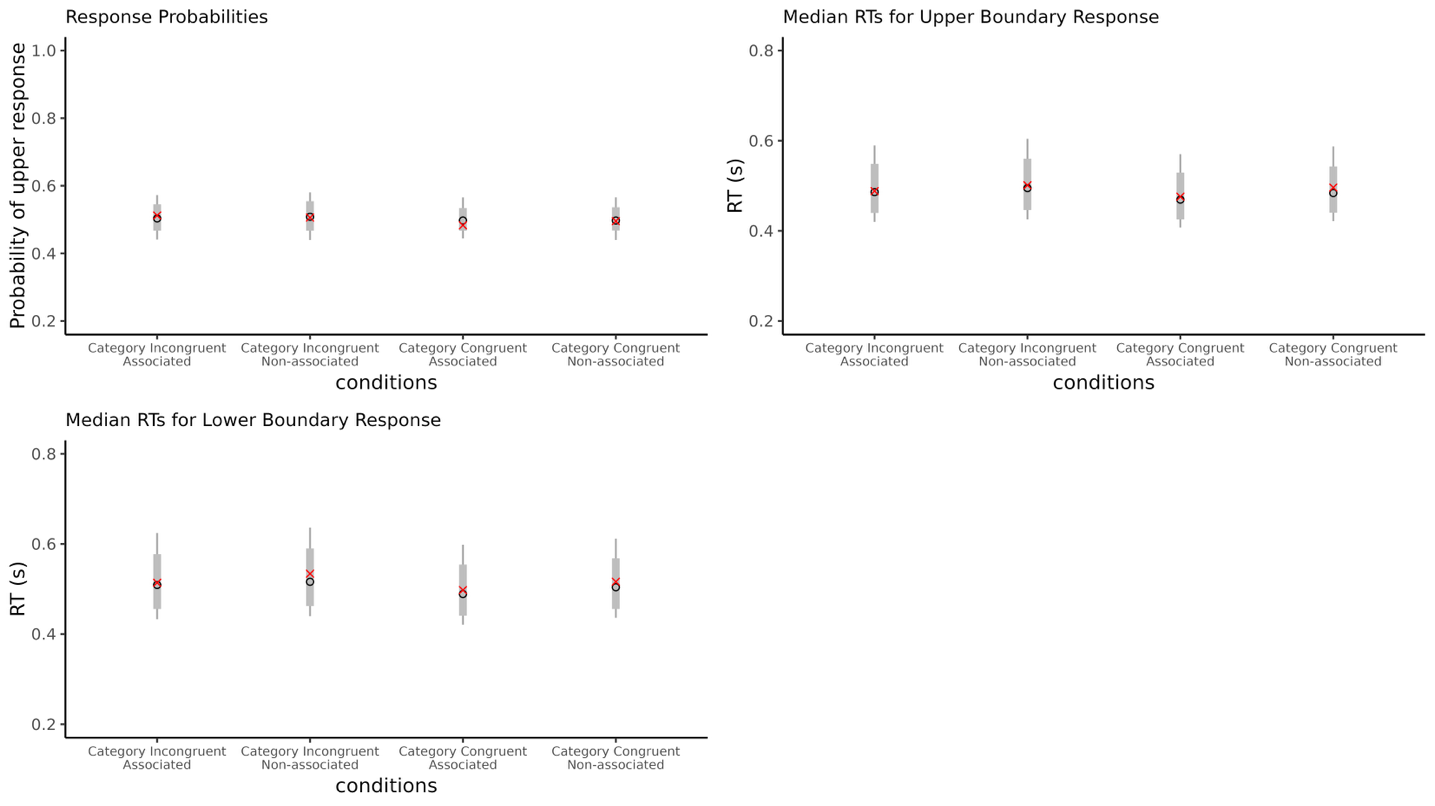


*Note. The red cross represents the median of the observed data and the black circle represents the median of the model prediction in each condition. The outer grey vertical lines indicate the 95% credible intervals, and the inner grey vertical lines indicate the 90% credible intervals of the model prediction.*

**Figure S3. Posterior predictive check (2) for model 8, comparing predicted and observed response times (RTs) across quantiles, separately for lower (non-living) and upper (living) responses.**


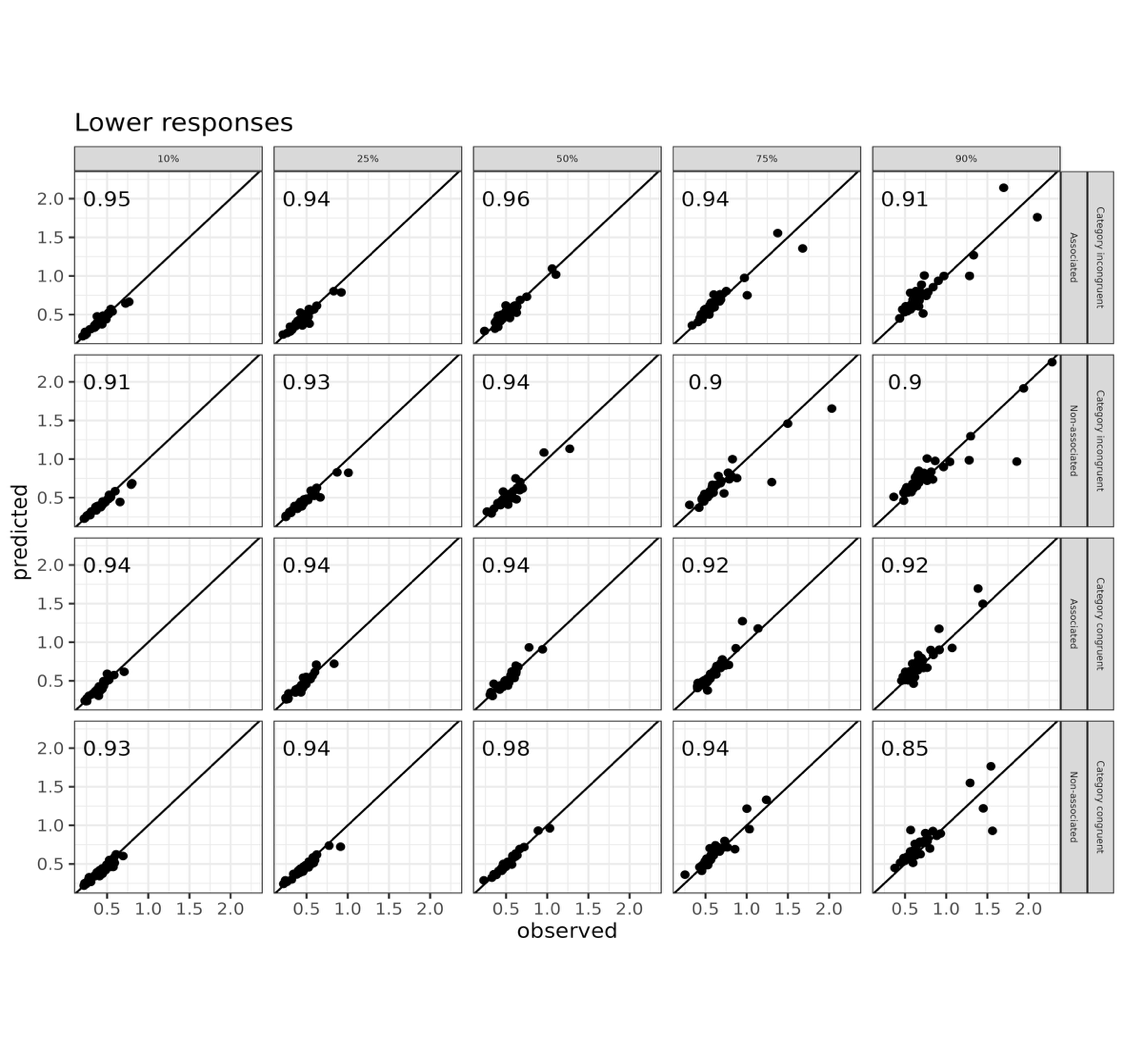


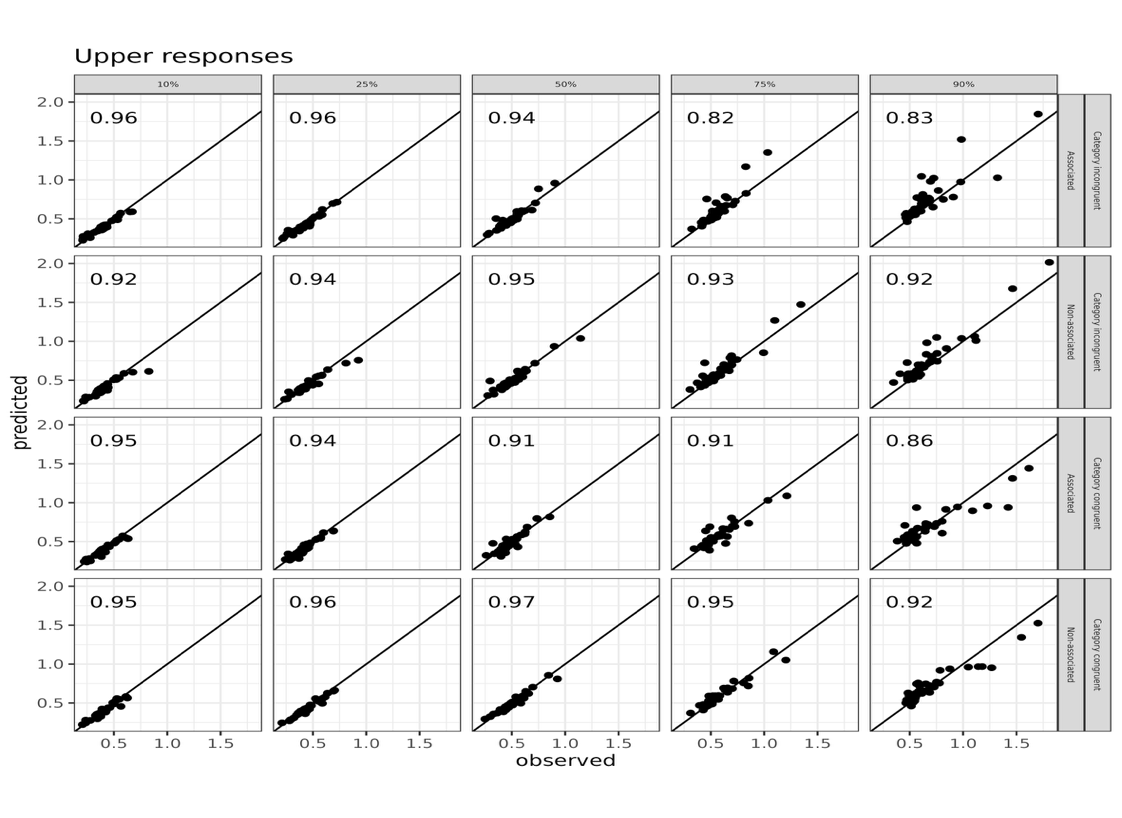


## Supplementary Material F: Random effects of hyperparameters in Model 8

Category congruence priming effect on drift rates showed a large variability across subjects. The posterior mean of $\sigma_{vc}$ was 0.438, 95% HDI was [0.343, 0.545]. The substantial individual differences were also found in drift rates $\sigma_{v}$ , mean = 1.580, 95% HDI = [1.338, 1.819]. Moreover, threshold variability among subjects was also relatively large, mean = 0.352, 95% HDI = [0.277, 0.436]. On the contrary, the component effects on non-decision times demonstrated minimal variability among participants.

Table S4 *Means, standard deviations, and 95% credibility intervals of posterior distributions of the random effects in Model 7.*

| Parameters | Mean | SD | Lower bound | Upper bound |
| --- | --- | --- | --- | --- |
| $\sigma_{a}$ | 0.352 | 0.041 | 0.277 | 0.436 |
| $\sigma_{w}$ | 0.046 | 0.007 | 0.032 | 0.060 |
| $\sigma_{v}$ | 1.580 | 0.126 | 1.338 | 1.819 |
| $\sigma_{t0}$ | 0.071 | 0.008 | 0.057 | 0.088 |
| $\sigma_{va}$ | 0.038 | 0.029 | 0.000 | 0.093 |
| $\sigma_{vc}$ | 0.438 | 0.052 | 0.343 | 0.545 |
| $\sigma_{vi}$ | 0.035 | 0.026 | 0.000 | 0.084 |
| $\sigma_{t0a}$ | 0.007 | 0.001 | 0.005 | 0.009 |
| $\sigma_{t0c}$ | 0.007 | 0.001 | 0.004 | 0.009 |
| $\sigma_{t0i}$ | 0.007 | 0.001 | 0.004 | 0.009 |

## Supplementary Material G: Effect course analysis

We calculated effect course analyses on RTs. Effect course analyses allow tracking how an effect develops throughout the course of an experiment. Hence, they could reveal how influences of the prime on target processing changed with practice. Following settings were chosen: Window size = 13, sample alpha = 0.1, and 5000 permutations were ran. Clusters were considered significant if p < .05.

Figure S4. Effect course analysis for RTs for the associative priming effect. Positive values suggest slower responses for non-associated compared to associated.


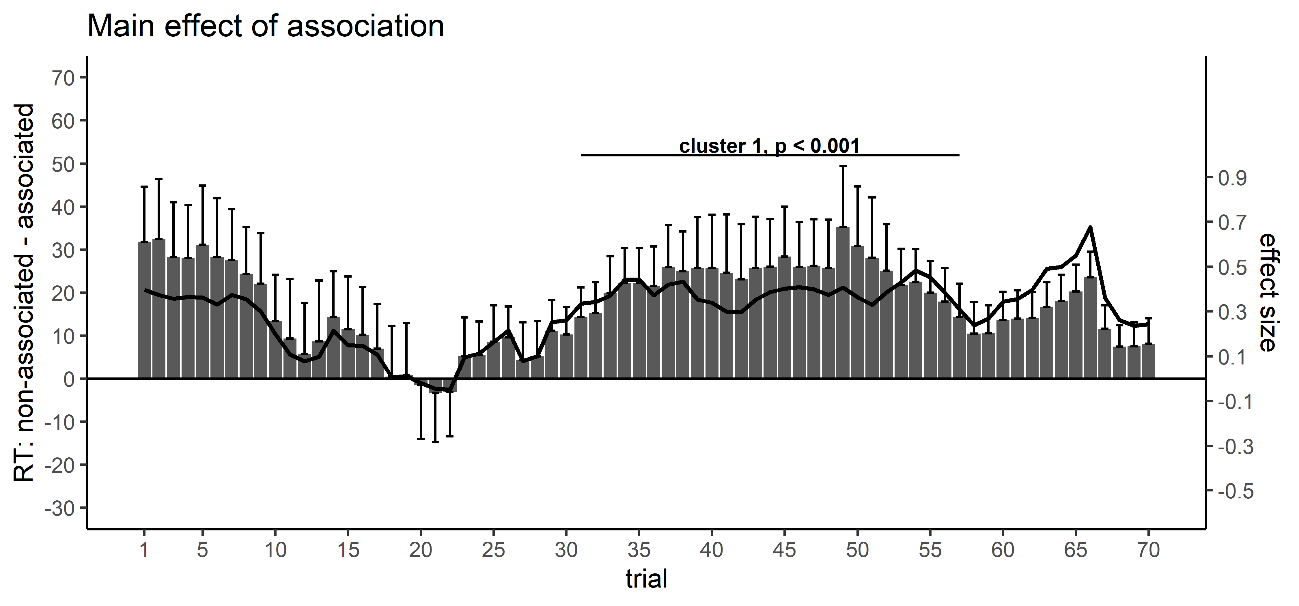


Figure S5. Effect course analysis for RTs for categorical priming effect. Positive values indicate slower response for category incongruent compared to category congruent targets.


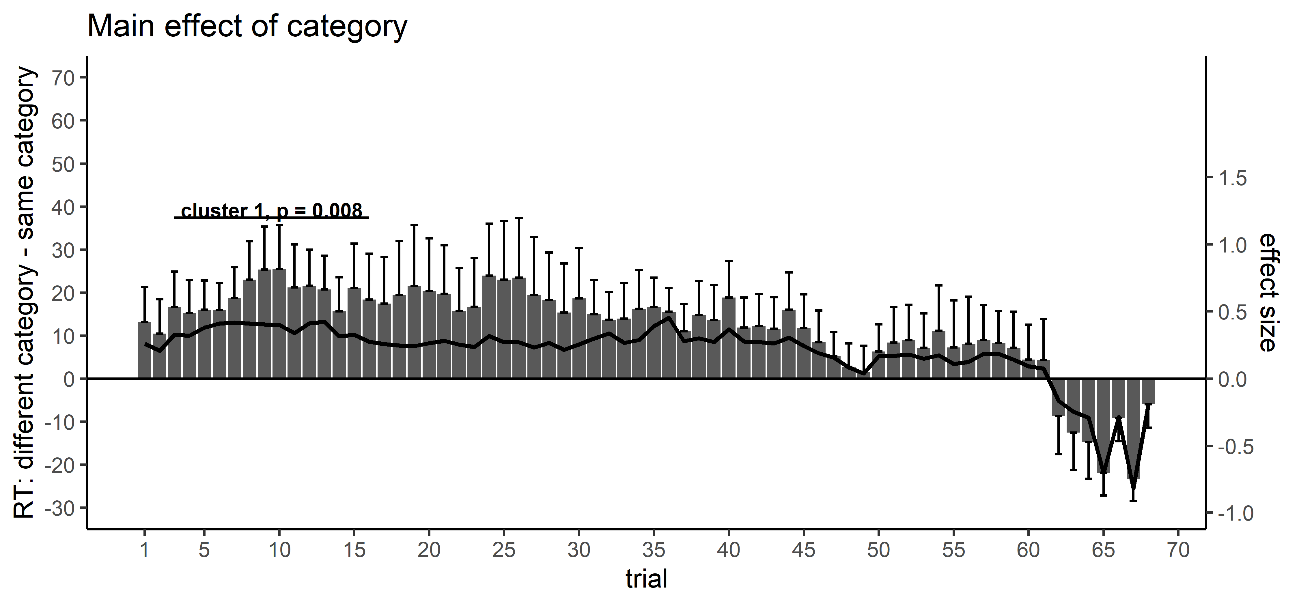


Figure S6 Effect course analysis for RTs for the living/non-living effect. Positive values indicate slower responses for non-living compared to living targets.


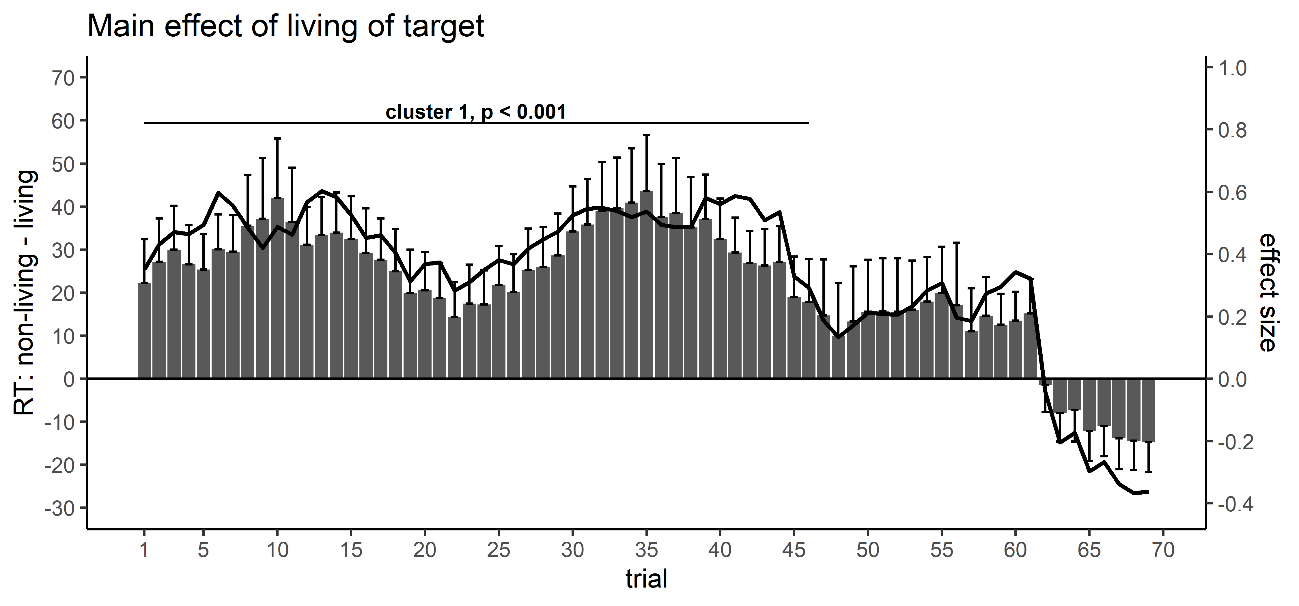

Supplement: Supplementary file 1 — Supplementary Material 1 (DOCX 6.90 MB) [file 426_2025_2234_MOESM1_ESM.docx]
